# Calcineurin associates with centrosomes and regulates cilia length maintenance

**DOI:** 10.1242/jcs.260353

**Published:** 2023-04-24

**Authors:** Eirini Tsekitsidou, Cassandra J. Wong, Idil Ulengin-Talkish, Angela I. M. Barth, Tim Stearns, Anne-Claude Gingras, Jennifer T. Wang, Martha S. Cyert

**Affiliations:** ^1^ Department of Biology, Stanford University, Stanford, CA 94305, USA; ^2^ Lunenfeld-Tanenbaum Research Institute, Mount Sinai Hospital, Toronto, ON, M5G 1X5, Canada; ^3^ Department of Genetics, Stanford School of Medicine, Stanford, CA 94305, USA; ^4^ Department of Molecular Genetics, University of Toronto, Toronto, ON, M5S 1A8, Canada

**Keywords:** Calcineurin, Phosphatase, POC5, Cilia, Centrosome, Centriole

## Abstract

Calcineurin, or protein phosphatase 2B (PP2B), the Ca^2+^ and calmodulin-activated phosphatase and target of immunosuppressants, has many substrates and functions that remain uncharacterized. By combining rapid proximity-dependent labeling with cell cycle synchronization, we mapped the spatial distribution of calcineurin in different cell cycle stages. While calcineurin-proximal proteins did not vary significantly between interphase and mitosis, calcineurin consistently associated with multiple centrosomal and/or ciliary proteins. These include POC5, which binds centrins in a Ca^2+^-dependent manner and is a component of the luminal scaffold that stabilizes centrioles. We show that POC5 contains a calcineurin substrate motif (PxIxIT type) that mediates calcineurin binding *in vivo* and *in vitro*. Using indirect immunofluorescence and ultrastructure expansion microscopy, we demonstrate that calcineurin colocalizes with POC5 at the centriole, and further show that calcineurin inhibitors alter POC5 distribution within the centriole lumen. Our discovery that calcineurin directly associates with centriolar proteins highlights a role for Ca^2+^ and calcineurin signaling at these organelles. Calcineurin inhibition promotes elongation of primary cilia without affecting ciliogenesis. Thus, Ca^2+^ signaling within cilia includes previously unknown functions for calcineurin in maintenance of cilia length, a process that is frequently disrupted in ciliopathies.

## Teaser

**Summary:** Calcineurin phosphatase participates in regulation of the centrosome and cilia. Calcineurin localizes to centrioles, where it interacts with POC5, and inhibition of calcineurin promotes elongation of cilia.

## INTRODUCTION

Ca^2+^ ions direct signaling within subcellular microdomains, where Ca^2+^-dependent effectors colocalize with their substrates (Ulengin-Talkish and Cyert, 2023). One such effector is calcineurin (CN; also known as protein phosphatase 2B, PP2B), a protein phosphatase that is activated by Ca^2+^ and calmodulin (CaM), and is inhibited by immunosuppressants, FK506 (also known as tacrolimus) and cyclosporin A (CysA) (Rusnak and Mertz, 2000). Because inhibiting CN in non-immune tissues has adverse consequences, systematically elucidating the substrates and subcellular distribution of CN is of clinical importance (Azzi et al., 2013).

CN, which is a heterodimer comprising a regulatory subunit (CNB; encoded by *PPP3R1* and *PPP3R2*) and a catalytic subunit (CNA; encoded by *PPP3CA*, *PPP3CB* and *PPP3CC*), recognizes substrates via two short linear peptide motifs (SLiMs) – degenerate sequences of 3–10 amino acid residues that mediate low-affinity, dynamic protein–protein interactions (Tompa et al., 2014). CN-binding SLiMs (LxVP and PxIxIT, where ‘x’ indicates any amino acid) have distinct properties: LxVP binding to active CN is required for dephosphorylation and is blocked by CN inhibitors. PxIxIT binding is independent of CN activation state, and anchors CN to substrates, regulators and scaffolds (Grigoriu et al., 2013; Roy and Cyert, 2020).

Identification of SLiMs is key to decoding CN signaling. Discovery of CN-binding SLiMs *in silico* has revealed hundreds of putative CN substrates (Wigington et al., 2020; Brauer et al., 2019; Sheftic et al., 2016). Proximity-dependent biotinylation coupled to mass spectrometry (PDB–MS) with wild-type (WT) or mutant CNA fused to a promiscuous biotin ligase has been used to identify SLiM-dependent CN interactions *in vivo* and to map CN-proximal proteins to multiple cellular compartments, including centrosomes, where the function of CN is unknown (Ulengin-Talkish et al., 2021; Wigington et al., 2020).

Centrosomes – microtubule-organizing centers containing two centrioles and associated pericentriolar material (PCM) – nucleate microtubules, form the mitotic spindle and direct formation of primary cilia (Carvalho-Santos et al., 2011). Primary cilia are non-motile sensory organelles, disruption of which results in disorders known as ciliopathies (Delling et al., 2013; Hildebrandt et al., 2011).

Here, we investigate the association of CN with centrosomes by mapping CN-proximal proteins using miniTurbo, a biotin ligase with a short labeling time (Branon et al., 2018), which allowed us to probe the subcellular distribution of CN throughout the cell cycle. We find that CN-proximal proteins do not change dramatically between interphase and mitosis but are significantly enriched for centrosomal proteins including POC5, which contains a CN-binding PxIxIT motif. Furthermore, CN colocalizes with POC5 at centrioles, and CN inhibition alters the centriolar distribution of POC5. Finally, we discover that CN inhibition promotes elongation of primary cilia without affecting ciliogenesis. Taken together, our findings establish new roles for CN and Ca^2+^ signaling at centrosomes and cilia.

## RESULTS AND DISCUSSION

### Subcellular CN distribution across the cell cycle

To identify CN-proximal proteins, CNAα (encoded by *PPP3CA*) was fused to miniTurbo, a promiscuous biotin ligase that labels proteins within a ∼10 nm radius in 15 min (Branon et al., 2018; Gingras et al., 2019). HEK293 cell lines overexpressing miniTurbo–3×FLAG alone or miniTurbo–3×FLAG fused to either WT CNAα (CNAα_WT_) or a mutant CNAα with impaired PxIxIT docking (CNAα_NIRmut_; Li et al., 2007) were incubated with biotin and analyzed by indirect immunofluorescence to assess biotin labeling and distribution (Fig. S1A–C).

**Fig. 1.**
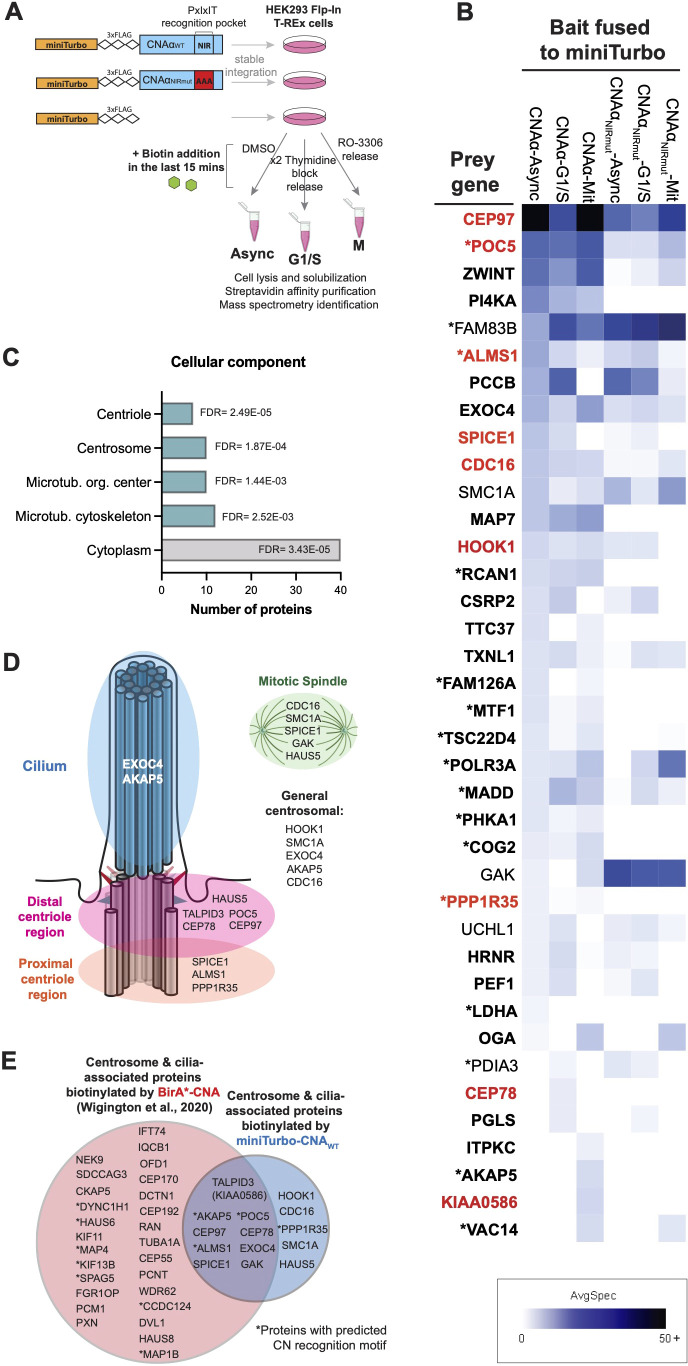
**Calcineurin is proximal to centrosome proteins.** (A) Scheme for cell cycle synchronization and PDB–MS. Amino acid residues mutated in the PxIxIT recognition pocket are indicted. Async, asynchronous; M, mitosis. (B) Heat map showing average spectral counts (AvgSpec) of the CN-proximal proteins (from DDA mass spectrometry) in each of the indicated experimental conditions. Proteins with PxIxIT docking-dependent biotinylation [log_2_ spectral count ratio (CNAα_WT/_CNAα_NIRmut_)≥0.5] are shown in bold type. Proteins annotated by the GO term ‘centrosome’ are shown in red. Asterisks indicate proteins containing PxIxIT or LxVP motifs. Mit, mitosis. (C) Cellular component GO terms with statistically significant enrichment among the CN-proximal proteins. The number of CN-proximal proteins assigned to each term is plotted, and the FDR is indicated. (D) CN-proximal proteins with previously reported localization to centrosomes, cilia or mitotic spindles. See Table S2 for references. (E) Overlap of centrosome and cilia CN-proximal proteins from Wigington et al. (2020) (pink circle) and this study (blue).

Next, to map the distribution of CN across the cell cycle, biotin-labeled cell populations that were asynchronous or synchronized either at the G1-S transition (G1/S) or in mitosis (Fig. 1A; Fig. S1D–H) were analyzed using mass spectrometry. Forty-one proteins were significantly biotinylated by CNAα_WT_ in at least one condition as identified through data-dependent acquisition (DDA) and data-independent acquisition [DIA; coupled with mixture-spectrum partitioning using libraries of identified tandem mass spectra (MSPLIT) analysis] mass spectrometry (38 proteins from DDA plus three additional proteins from MSPLIT–DIA; Fig. 1B; Fig. S1I, Table S1). We found that these proteins are enriched for protein–protein interactions (Fig. S1J) and include known CN interactors and substrates: AKAP5 (Dell'Acqua et al., 2002), PI4KA, FAM126A (also known as HYCC1; Ulengin-Talkish et al., 2021), PHKA1 (Ingebritsen and Cohen, 1983) and RCAN1 (Mehta et al., 2009). Biotinylation of most of the CN-proximal proteins was PxIxIT docking dependent (35/41 proteins), and 17 of the proteins contained a predicted CN-specific SLiM (Fig. 1B; Fig. S1I, Table S1). Fewer CN-proximal proteins were identified than in a previous study (Wigington et al., 2020), likely due to decreased labeling time (15 min versus 18 h), and 15 proteins were common to both datasets (Table S1).

Notably, the most robustly detected CN-proximal proteins were common to all three conditions (23/41 proteins). Proteins detected only in mitosis (4/41 proteins) or G1/S (3/41 proteins) were represented by low spectral counts (≤7), possibly at the limit of detection, and had no reported cell-cycle-specific functions. Thus, CN spatial distribution seems relatively constant through the cell cycle, with signaling dictated by spatiotemporally regulated Ca^2+^ transients.

### CN proximity to centrosomal components

Interestingly, the CN-proximal proteins were significantly enriched for the gene ontology (GO) categories ‘centrosomes’, ‘centrioles’ (Fig. 1B,C; Table S1), and the ‘cilium assembly’ pathway [false discovery rate (FDR) 0.044] (Mi et al., 2013). Furthermore, a manually curated literature search identified that 14 of the CN-proximal proteins localize throughout the centrosome, cilium and/or mitotic spindle (Fig. 1D; Table S2); nine of these proteins have previously been identified as CN-proximal proteins (Fig. 1E; Wigington et al., 2020).

Thus, CN may contact centrosomal proteins, either at centrosomes or before their incorporation into centrosomes. CN proximity to mitotic spindle proteins suggests that CN may function during mitosis, when centrosomal Ca^2+^ signals have been observed (Helassa et al., 2019).

### CNB localizes to the centrosome

**Fig. 2.**
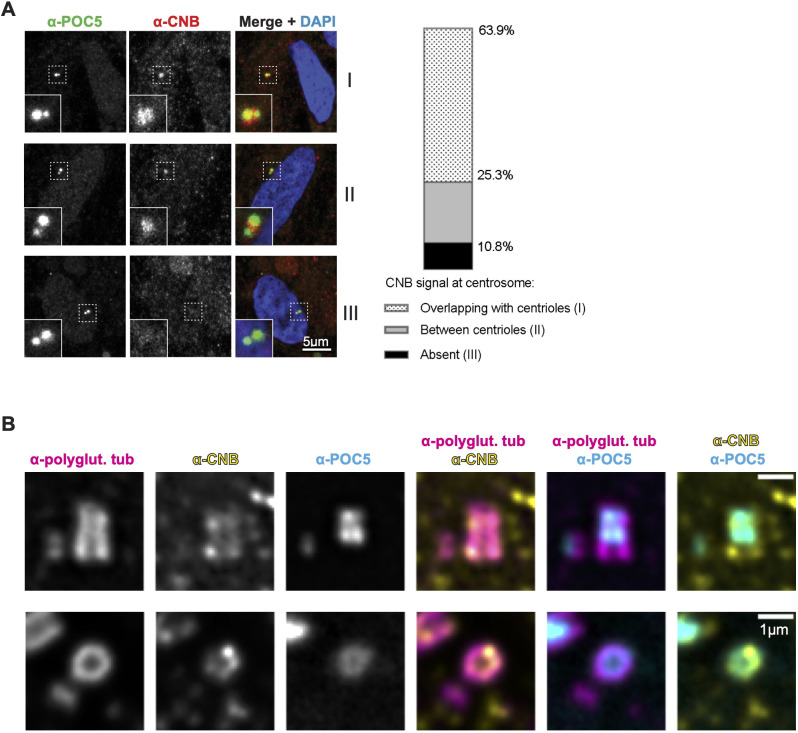
**CNB localizes to centrosomes.** (A) Immunofluorescence staining of POC5 and CNB in cytosol-depleted hTERT-RPE1 cells. Left: maximum-intensity projections of confocal *z*-stacks representing the three categories of CNB localization observed (I, II and III). Dashed boxes indicate regions enlarged in the inset images. Right: bar chart showing the CNB localization pattern frequency in each of the three indicated categories. *n*=83 cells. (B) Deconvolved U-ExM images of centrosomes from hTERT-RPE1 cells, stained for polyglutamylated tubulin (polyglut. tub), CNB and POC5. Top: longitudinal view of parental centriole. Bottom: cross-section view of parental centriole. Single *z*-plane images. Images are representative of 25 cells.

Next, we examined whether CN localizes to centrosomes by using indirect immunofluorescence of hTERT-RPE1 cells permeabilized with digitonin prior to fixation (Sydor et al., 2018). Independent of cell cycle phase, CNB, which always associates with CNA, was observed surrounding (63.9%) or distributed between (25.3%) centrioles, or was not centriole-associated (10.8%) (Fig. 2A). Centrosomal CNB staining was specific; it was eliminated by pre-incubating anti-CNB antisera with purified CNB but not by pre-incubating with bovine serum albumin (BSA; Fig. S2A,B).

To examine centrosomal CNB localization more precisely, we performed ultrastructure expansion microscopy (U-ExM) (Gambarotto et al., 2019) with the anti-CNB antiserum used for the experiment in Fig. 2A. Despite increased background signal caused by cytoplasmic CNB, CNB was observed to consistently colocalize with polyglutamylated tubulin at the microtubule walls of centriole barrels, close to POC5 (Fig. 2B). These results provide the first evidence that a pool of CN specifically localizes to centrioles, where it may interact directly with centriolar proteins.

### CN–POC5 interaction is PxIxIT dependent

**Fig. 3.**
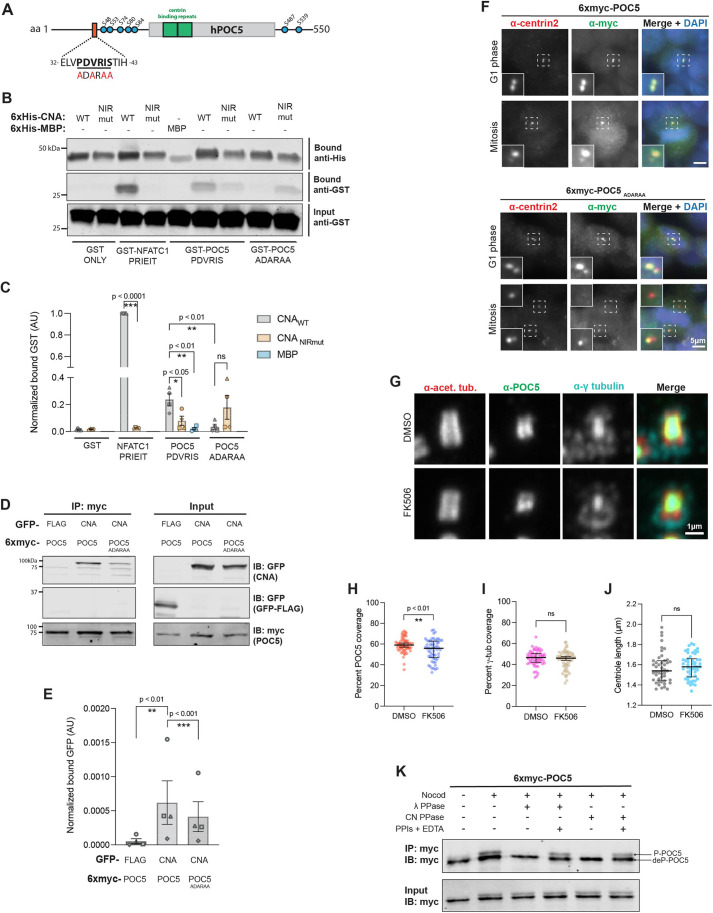
**Calcineurin interacts with POC5 in a PxIxIT-dependent manner.** (A) Schematic of human POC5 (hPOC5; UniProt ID Q8NA72-3). Orange, PxIxIT motif with WT (PDVRIS) and mutant (ADARAA) sequences shown beneath; green, centrin-binding repeats; blue circles, phosphorylated residues (from PhosphoSitePlus database; Hornbeck et al., 2015). (B) POC5 contains a CN-binding PxIxIT motif. Co-purification of GST-tagged PxIxIT peptides from NFATC1 and POC5, as indicated, with 6×His–MBP, 6×His–CNAα_WT_:CNB or 6×His–CNAα_NIRmut_:CNB. (C) Quantification of the experiment shown in B (bound GST signal/bound His signal, normalized to input GST signal; AU, arbitrary units). Data are mean±s.e.m. (*n*=4 independent experiments). ns, not significant; *P<0.05; **P<0.01; ***P<0.001 (unpaired, two-tailed Student's *t*-test). (D) CN association with POC5 is PxIxIT-dependent *in vivo*. GFP–FLAG or GFP–CNAα_WT_ co-immunoprecipitated with 6×myc–POC5_WT_ or 6×myc–POC5_ADARAA_ expressed in HeLa cells. IB, immunoblot; IP, immunoprecipitation. (E) Quantification of the experiment shown in D (bound GFP signal/bound myc signal, normalized to input GFP signal; AU, arbitrary units). Data are mean±s.e.m. (*n*=4 independent experiments). **P<0.01; ***P<0.001 (ratio-paired, two-tailed *t*-test). (F) Transiently expressed 6×myc–POC5_WT_ (top) or 6×myc–POC5_ADARAA_ (bottom) is recruited to centrosomes in HeLa cells. Single *z*-plane images of cells stained for centrin 2 and myc-tagged POC5 in the indicated cell cycle stages are shown (representative of 100 cells). Dashed boxes indicate regions magnified in inset images. (G) U-ExM of centrosomes in hTERT-RPE1 cells treated with DMSO or 2.5 μM FK506 for 48 h. Maximum-intensity projections of confocal *z*-stacks showing staining of acetylated tubulin (acet. tub.), POC5 and γ-tubulin. (H) CN inhibition decreases POC5 luminal distribution. Percentage of centriole covered by POC5 is plotted. (I) CN inhibition does not alter γ-tubulin (γ-tub) luminal distribution. Percentage of centriole covered by γ-tubulin is plotted. In H and I, data are pooled from two independent experiments, with individual data points plotted and the median±interqurtile range (IQR) indicated. DMSO, *n*=60 centrioles; FK506, *n*=58 centrioles. ns, not significant; **P<0.01 (unpaired, two-tailed Student's *t*-test). (J) CN inhibition does not alter centriole length. Median±IQR centriole length is shown. Data pooled from two independent experiments. DMSO, *n*=46 centrioles; FK506, *n*=51 centrioles. ns, not significant (unpaired, two-tailed Student's *t*-test). (K) CN dephosphorylates mitotic POC5 *in vitro*. Immunoblots of *in vitro* dephosphorylation of 6×myc–POC5 by λ phosphatase or CN. Nocod, nocodazole synchronization; λ PPase, λ phosphatase; CN PPase, purified constitutively active truncated 6×His–CNAα_WT_:CNB; PPIs, phosphatase inhibitors; P-POC5, phosphorylated POC5; deP-POC5, dephosphorylated POC5. Blots shown are representative of three experiments.

To identify CN-binding partners at centrioles, we focused on POC5, which showed PxIxIT docking-dependent biotinylation (Fig. 1B; Table S1) and contains a predicted PxIxIT motif (Fig. 3A) (Wigington et al., 2020). POC5 is required for centriole maturation and ciliogenesis, and is part of a luminal scaffold that stabilizes the centriole microtubule barrel (Azimzadeh et al., 2009; Le Guennec et al., 2020). POC5 mutations cause adolescent idiopathic scoliosis (Hassan et al., 2019) and retinitis pigmentosa (Weisz Hubshman et al., 2018).

To investigate whether CN recognizes the predicted POC5 PxIxIT motif (Fig. 3A), WT and mutant POC5 peptides were fused to GST and tested for co-purification with constitutively active truncated His-tagged WT CN (CN_WT_; 6×His–CNAα_WT_:CNB) and mutant CN (CN_NIRmut_; 6×His–CNAα_NIRmut_:CNB) *in vitro*. CN_WT_ co-purified with PxIxIT peptides from POC5 (PDVRIS) and NFATC1, but not with GST alone. CN_WT_ co-purification was significantly reduced with the mutant POC5 peptide (ADARAA), and CN_NIRmut_ co-purified weakly with all peptides (Fig. 3B,C), consistent with PxIxIT-mediated binding.

To examine CN interaction with full-length POC5, GFP–CNAα_WT_ or GFP–FLAG was co-expressed with myc-tagged WT or PxIxIT-mutant POC5 (6×myc–POC5_WT_ and 6×myc–POC5_ADARAA_, respectively) in HEK293T cells. GFP–CNAα_WT_ (but not GFP–FLAG) co-immunoprecipitated with POC5_WT_ and showed reduced interaction with POC5_ADARAA_ (Fig. 3D,E), indicating that full-length POC5 binds directly to CN through the PxIxIT motif.

Next, we sought to identify a function for POC5–CN interaction. To examine whether the POC5 PxIxIT motif is required for centriolar recruitment, we carried out indirect immunofluorescence of HeLa cells transiently expressing 6×myc–POC5 (POC5_WT_ or mutant POC5_ADARAA_) in the presence of endogenous POC5. Both proteins colocalized with centrin 2 at centrioles in G1 phase and at spindle poles in mitosis (Fig. 3F). Although these analyses revealed no CN dependence, we reasoned that qualitative studies of overexpressed POC5 might not be sufficiently sensitive.

Therefore, using U-ExM, we examined the distribution of endogenous POC5 and γ-tubulin in the centriolar lumen of hTERT-RPE1 cells in the presence or absence of the CN inhibitor FK506 (Fig. 3G), which does not alter cell cycle progression (Fig. S3A,B). POC5 is required for γ-tubulin recruitment to the centriole lumen but not to the PCM (Schweizer et al., 2021). Coverage of the centriole lumen by POC5, but not by γ-tubulin, was significantly reduced in centrioles from interphase and mitotic cells in which CN was inhibited (Fig. 3H,I; Fig. S3C). No other changes to centriole morphology or length were observed (Fig. 3J). Thus, CN activity may modify POC5 association with or distribution within the centriole lumen, while not disrupting recruitment of γ-tubulin.

Taken together, these findings show that CN interacts directly with POC5 via a PxIxIT motif, and that CN activity affects POC5 distribution within the centriole lumen. Further analyses are required to examine whether CN–POC5 binding is required for centriolar POC5 distribution and/or CN localization to centrosomes.

### CN dephosphorylates POC5 *in vitro*

We hypothesized that CN modifies POC5 distribution through dephosphorylation. The function of POC5 phosphorylation, some of which is mitotic, is unknown (Fig. 3A) (Azimzadeh et al., 2009). To test whether CN dephosphorylates POC5, 6×myc–POC5_WT_ was immunopurified from mitotic HeLa cells and analysed by immunoblotting. The purified POC5 was observed to form a doublet, with phosphorylated POC5 (p-POC5) migrating more slowly (Fig. 3K, input). Incubating POC5 *in vitro* with constitutively active truncated 6×His–CNAα_WT_:CNB or the non-specific λ phosphatase eliminated p-POC5, which was preserved when phosphatase inhibitors were included (Fig. 3K). However, in extracts of HeLa cultures expressing 6×myc–POC5_wT_ or 6×myc–POC5_ADARAA_ that were enriched for mitotic cells (Fig. S3D–F), neither activation (ionomycin and Ca^2+^) nor inhibition (FK506) of CN altered POC5 electrophoretic migration (Fig. S3G,H). POC5 is also hyperphosphorylated in nuclear/centrosomal fractions of asynchronous cells (Azimzadeh et al., 2009), but this POC5 population also failed to show Ca^2+^- or CN-dependent changes in electrophoretic mobility (Fig. S3G,I).

Thus, although our findings that CN interacts with POC5, modifies POC5 distribution within the centriole lumen and dephosphorylates POC5 *in vitro* suggest that POC5 is a CN substrate, we failed to obtain support for this hypothesis *in vivo*. This issue should be re-examined once the mechanisms and functional significance of POC5 phosphorylation have been elucidated.

### CN regulates the length of primary cilia

**Fig. 4.**
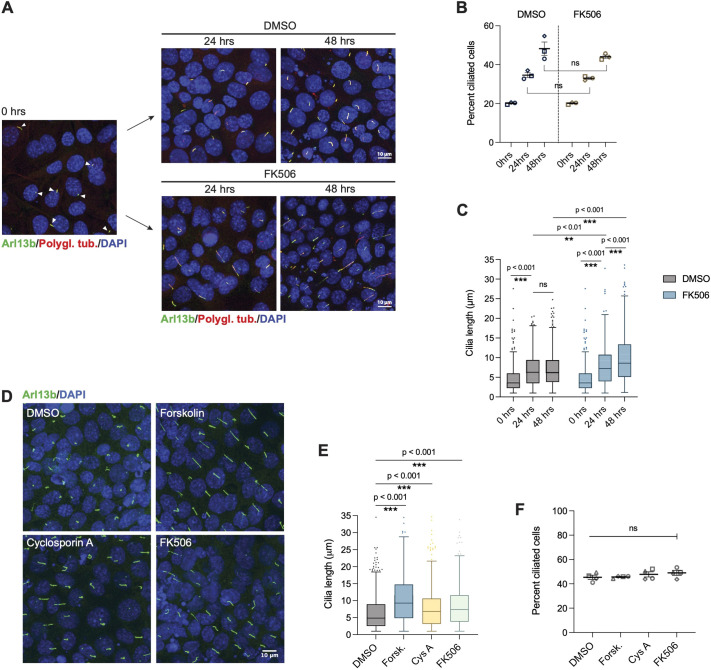
**Calcineurin inhibition promotes elongation of cilia.** (A) CN inhibition increases cilia length without disrupting ciliogenesis. IMCD3 cell cilia after the indicated treatments with DMSO or 2.5 μM FK506. Cells are stained to show Arl13b and polyglutamylated tubulin (polygl. tub.). White arrowheads indicate short cilia. Images are maximum-intensity projections. (B) Percentage ciliated cells quantified using images as shown in A. Data are presented as the mean±s.e.m. of *n*=3 independent experiments. Total *n* cells: 0 h, *n*=1319; 24 h DMSO, *n*=2176; 24 h FK506, *n*=2468; 48 h DMSO, *n*=3710; 48 h FK506, *n*=4120. ns, not significant, *P*>0.05 (unpaired, two-tailed Student's *t*-test. (C) Length of cilia quantified using images as shown in A. Boxplots show the median (line) and interquartile range (IQR; box), with Tukey whiskers indicating the 75th percentile+1.5×IQR and 25th percentile−1.5×IQR. Data pooled from three independent experiments. Total *n* cilia: 0 h, *n*=331; 24 h DMSO, *n*=556; 24 h FK506, *n*=256; 48 h DMSO, *n*=931; 48 h FK506, *n*=429. ns, not significant; **P<0.01; ***P<0.001 (two-tailed Mann–Whitney test). (D) Forskolin and CN inhibition increase cilia length but not number. IMCD3 cell cilia treated with DMSO, 10 μM forskolin or CN inhibitors (2.5 μM FK506; 2.5 μM cyclosporin A), as indicated, for 3 h. Images are maximum-intensity projections. (E) Length of cilia quantified using images as shown in D. Boxplots show the median (line) and IQR (box), with Tukey whiskers indicating the 75th percentile+1.5×IQR and 25th percentile−1.5×IQR. Data shown for one of four replicates; additional replicates are shown in Fig. S4. Total *n* cilia: DMSO, *n*=537; forskolin, *n*=514; cyclosporin A, *n*=553; FK506, *n*=638 cilia. ***P<0.001 (two-tailed Mann–Whitney test). (F) Percentage ciliated cells quantified using images as shown in D. Data are presented as the mean±s.e.m. of *n*=4 independent experiments. *n*>1200 cells per replicate per treatment. ns, not significant, *P*>0.05 (unpaired, two-tailed Student's *t*-test).

Because CN-proximal proteins (such as POC5 and CEP97) and CN-binding partners (CaM, nucleoporins and RCAN2) are required for cilia function (Schweizer et al., 2021; Spektor et al., 2007; Kee et al., 2012; Plotnikova et al., 2012; Stevenson et al., 2018), we asked whether CN affects ciliogenesis. IMCD3 cells were induced to ciliate via contact inhibition (Joly et al., 2006) and simultaneously were treated with DMSO (control) or FK506 (Fig. 4A,B). After 48 h, ∼50% of control or CN-inhibited cells were ciliated (Fig. 4B), indicating that CN is not required for ciliogenesis. However, cilia were significantly longer upon CN inhibition. Under control conditions, cilia length at 24 h (median=6.27 μm) was maintained over the next 24 h. In contrast, cilia in CN-inhibited cells were longer at 24 h (median=7.23 μm) and continued to grow (48 h, median=8.59 μm; Fig. 4C).

Previous studies have shown that Ca^2+^ and protein kinase A (PKA) signaling antagonistically regulate the length of cilia, and that acutely activating adenylyl cyclase with forskolin, or inhibiting Ca^2+^ entry, both lengthen cilia by increasing PKA activity (Besschetnova et al., 2010). To examine the role of CN in maintaining cilia length, we treated confluent, ciliated IMCD3 cells with CN inhibitors for 3 h, which resulted in significant lengthening of cilia relative to those of control cells, although less than was observed following forskolin treatment (Fig. 4D,E; Fig. S4). None of these treatments altered the proportion of ciliated cells (Fig. 4F), suggesting that CN regulates cilia length maintenance rather than cilium assembly.

In sum, our results reveal CN localization to centrioles and suggest that CN modifies one or more aspects of centriole and/or cilia homeostasis. Most previous research has focused on the regulation of centrosomes by kinases, but our findings highlight roles for phosphatases and Ca^2+^ signaling at these organelles. Previously observed centrosomal Ca^2+^ signals (Helassa et al., 2019) activate downstream effectors such as CaM (Plotnikova et al., 2012) and centrins, whose Ca^2+^ binding is required for centrosomal localization and interaction with POC5 (Khouj et al., 2019). Our finding that CN binds directly to POC5 and alters its centriolar distribution suggest direct regulation of this protein, despite our inability to detect CN-dependent dephosphorylation *in vivo*. POC5 promotes ciliogenesis by recruiting augmin and γ-tubulin ring complexes (γ-TuRCs) to the centriole lumen (Schweizer et al., 2021). Of eight augmin subunits, HAUS5, HAUS6 and HAUS8 are CN-proximal proteins, with two PxIxIT motifs predicted within HAUS6 (Wigington et al., 2020). Thus, potential regulation of centriolar stability and/or ciliary function via dephosphorylation of POC5, augmin or other centriolar components by CN warrants further investigation.

We have also discovered that CN regulates the length of cilia, through mechanisms that remain to be determined. Our findings are consistent with the previous observation that depletion of RCAN2, a negative regulator of CN that localizes to centrioles and basal bodies, causes ciliary shortening (Stevenson et al., 2018). CN might regulate cilia length via POC5 or other structural proteins. Alternatively, CN might contribute to Ca^2+^-dependent antagonism of cAMP–PKA signaling (Besschetnova et al., 2010), perhaps via AKAP5, which is a scaffold for CN and PKA that localizes to centrosomes and primary cilia, where it associates with adenylyl cyclases AC5 and AC6 (ADCY5 and ADCY6, respectively) and the polycystin-2 (PC2) Ca^2+^ channel (Choi et al., 2011). Remarkably, CN targets PC2 to cilia in *Caenorhabditis elegans* (Hu et al., 2006), although this has not been investigated in mammals. In ciliopathies, cilia length is frequently disrupted in highly ciliated organs, such as the kidney and retina (Moreno-Leon et al., 2021; Veland et al., 2009). Thus, elucidating mechanisms through which CN regulates cilia length and centriolar function promises to improve our current understanding of both CN signaling and ciliopathies.

## MATERIALS AND METHODS

### Cell lines and culture

Cells were cultured at 37°C in 5% CO_2_. HEK293T, HeLa and mouse IMCD3 cells were grown in Dulbecco's Modification of Eagle's Medium (DMEM) with 4.5 g/l glucose, L-glutamine and sodium pyruvate (Corning, 10-013-CV) supplemented with 10% fetal bovine serum (FBS; Benchmark^TM^, Gemini Bio Products, 100-106). HEK293T and HeLa cells were a gift from Jan Skotheim's lab at Stanford University, CA, USA. IMCD3 cells were a gift from Peter Jackson's lab at Stanford University. hTERT-RPE1 cells (ATCC, CRL-4000) were cultured in DMEM/F12 medium (Gibco, 11320033) supplemented with 10% FBS. Parental HEK293 Flp-In T-REx cells (Invitrogen, R78007) were cultured in DMEM supplemented with 10% FBS, 3 μg/ml blasticidin (Research Products International, B12150) and 100 μg/ml Zeocin (Gibco, R25001) prior to stable plasmid integration. Mycoplasma testing was conducted monthly using a mycoplasma PCR detection kit (ABM, G238). Human and mouse cell lines were authenticated by short tandem repeat (STR) profiling (Almeida et al., 2019; Barallon et al., 2010). IMCD3 cells were authenticated using the ATCC Mouse Cell Authentication Service (ATCC, 137-XV).

### MiniTurbo–3×FLAG stable cell lines

MiniTurbo–3×FLAG constructs were generated via Gateway cloning into pDEST 5′ miniTurbo–3×FLAG pcDNA5 FRT TO, which was constructed as described previously (Rosenthal et al., 2021) but using miniTurbo sequences optimized for expression in mammalian cells. MiniTurbo–3×FLAG-CNAα contained human PPP3CA amino acids 1–521, using either the WT sequence (CNAα_WT_) or sequence with residues NIR (amino acids 330–332) mutated to AAA (CNAα_NIRmut_). Stable cell lines were generated in HEK293 Flp-In T-REx cell pools as described previously for BirA*–FLAG (Hesketh et al., 2017). Doxycycline-inducible, miniTurbo-expressing HEK293 Flp-In T-REx cells were cultured in antibiotic selection medium: DMEM supplemented with 10% FBS, 3 μg/ml blasticidin and 200 μg/ml hygromycin B (Research Products International, H75000-1). MiniTurbo expression was induced with addition of 1 μg/ml doxycycline (Sigma-Aldrich, D9891) for 48 h. To visualize biotin distribution in miniTurbo cell lines, asynchronous cells were induced and treated with 50 μM D-biotin (Bio Basic, BB0078) for 15 min at 37°C. Cells were imaged via indirect immunofluorescence with an antibody against centrin 2 (see Antibodies section below), as well as Alexa Fluor 594-conjugated streptavidin (1:1000; Thermo Fisher Scientific, S-11227). Microscopy images were acquired as *z*-stacks collected at 0.50 μm intervals using a widefield Lionheart FX automated microscope (Agilent/BioTek) with an Olympus Plan Fluorite 0.7 NA 60× air objective and a Sony CMOS 16-bit grayscale camera. Gen5 Software (BioTek) was used to control the microscope system. ImageJ (NIH, Bethesda, MD, USA) was used for image analysis. During proximity-dependent biotinylation assays, stable cell lines were cultured in DMEM supplemented with 10% FBS previously treated to remove residual biotin (see below), in order to reduce the possibility of non-specific biotinylation.

### Plasmid transfection

For stable cell line generation, HEK293 Flp-In T-Rex cells were co-transfected with pOG44 Flp-Recombinase expression vector (Invitrogen, V600520), and pcDNA5 FRT TO plasmids expressing appropriate miniTurbo gene fusions, using Lipofectamine™ 2000 (Invitrogen, 11668027), according to the manufacturer's instructions. All other plasmid transfections were done using jetOPTIMUS DNA Transfection Reagent (Polyplus), according to the manufacturer's instructions.

### Biotin depletion of FBS

To remove residual biotin from serum for proximity-dependent biotinylation assays, streptavidin Sepharose high-performance beads were used (Cytiva, 17-5113-01). 50 μl of packed bead volume was rinsed three times with phosphate-buffered saline (PBS, pH 7.4; Gibco 10010049) in sterile conditions. Beads were spun at 500 ***g*** for 1 min to remove the supernatant, then resuspended in a volume of PBS equal to the bead volume, resulting in a 1:1 bead:PBS ratio. Resuspended beads were added to 50 ml of FBS and allowed to mix at 4°C for 3 h. The serum was then spun at 1000 ***g*** for 5 min to pellet the beads, and the supernatant was filtered through a syringe attached to a 0.45 μm low-bind filter under sterile conditions.

### Cell cycle synchronization coupled to biotinylation

For the asynchronous cell population, miniTurbo–3×FLAG HEK293 Flp-In T-Rex cells were cultured in DMEM containing 10% biotin-depleted FBS, 1 μg/ml doxycycline and DMSO for 48 h. Immediately prior to cell collection, 50 μM D-biotin (Bio Basic, BB0078) was added to the medium for 15 min at 37°C. For G1/S synchronization, cells were cultured in DMEM containing 10% biotin-depleted FBS and 1 μg/ml doxycycline on day 1. On day 2, 2.5 μM thymidine (Millipore-Sigma, T9250) was added to the medium for 14 h at 37°C. Cultures were then rinsed with PBS, and fresh medium with 10% biotin-depleted FBS and 1 μg/ml doxycycline was added for 10 h at 37°C. 2.5 μM thymidine was then added to the medium for 24 h at 37°C. Cultures were again rinsed with PBS. Fresh medium with 10% biotin-depleted FBS, 1 μg/ml doxycycline and 50 μM D-biotin was added for 15 min immediately prior to cell collection. For mitotic synchronization, cells were cultured in DMEM containing 10% biotin-depleted FBS and 1 μg/ml doxycycline on day 1. On day 2, 9 μM RO-3306 (Selleck Chemicals, S7747) was added to the medium for 20 h at 37°C. Cells were then rinsed with PBS and incubated in DMEM with 10% biotin-depleted FBS and 1 μg/ml doxycycline at 37°C for 45 min. 50 μM D-biotin was then added to the medium for 15 min, and cells were finally collected 1 h after RO-3306 release. Cells were collected by the addition of warm Trypsin–EDTA Solution, 0.25% (Gibco™, 25200056), and a subset of them were kept resuspended in medium for further analysis by flow cytometry and immunoblotting. Collected cells were pelleted by centrifugation at 500 ***g*** for 5 min. Cell pellets were weighed, frozen in liquid nitrogen and stored at −80°C until further analysis. Each biotinylation experiment was performed twice, resulting in two biological replicates, or two cell pellets, per condition.

### Validation of proximity-dependent biotinylation samples by flow cytometry and immunoblotting

To prepare for flow cytometry, ∼1×10^6^ synchronized HEK293 Flp-In T-Rex cells were resuspended in 1 ml 3% paraformaldehyde (PFA) solution and incubated at 37°C for 10 min. Ice-cold methanol (at a volume ratio of 1:9 PFA:methanol) was then added dropwise to the cell suspension, which was incubated in ice for 30 min. Fixed cells were pelleted by centrifugation at 1000 ***g*** at 4°C for 10 min and the supernatant was removed. The remaining pellet was resuspended in 500 μl BSA (3% solution in PBS; Sigma-Aldrich, A3294) and centrifuged again at 1000 ***g*** at 4°C for 10 min. The supernatant was removed, and the pellet was resuspended in 500 µl of DAPI (Cayman Chemical, 14285; 20 μg/ml solution in PBS). The cells were incubated with DAPI at room temperature for 15 min, protected from light, and then analyzed for DAPI fluorescence (405 nm laser, VL-1 channel) using the Attune™ NxT Flow Cytometer (Invitrogen), until 50,000 cells had passed through the flow cytometer for each sample. Data were analyzed using the Attune™ NxT Software v3.1.2.

To validate bait expression and successful cell cycle synchronization, synchronized and induced HEK293 Flp-In T-Rex cells were pelleted by centrifugation at 500 ***g*** for 5 min, frozen in liquid nitrogen, thawed and lysed with RIPA buffer (50 mM Tris-HCl pH 8, 150 mM NaCl, 1% Triton X-100, 0.5% sodium deoxycholate and 0.1% SDS). Samples were resolved by SDS–PAGE and immunoblotted.

### Immunoblotting

For immunoblotting, samples were denatured with 2× or 6× SDS Laemmli buffer and incubated at 95°C for 5 min. Protein concentrations were determined using the Pierce BCA Protein Assay Kit (Thermo Fisher Scientific, 23225), according to the manufacturer's instructions. Equal amounts of protein (20–40 μg) were separated by SDS–PAGE. Proteins were transferred to a nitrocellulose membrane (Bio-Rad, 162-0112). The membrane was blocked with SuperBlock blocking buffer (Thermo Fisher Scientific, PI37535) at room temperature for 30 min and then incubated with primary antibodies at 4°C overnight, or at room temperature for 1 h. Next, the membrane was incubated with secondary antibodies at room temperature for 1 h. All blots were imaged with the LI-COR Odyssey imaging system and analyzed using Image Studio (LI-COR Biosciences). Images of uncropped immunoblots are shown in Figs S5 and S6.

### Biotin–streptavidin affinity purification and on-bead trypsin digest

Frozen cell pellets were first thawed and then lysed, bound to streptavidin Sepharose beads, trypsinized, dried and prepared for analysis by mass spectrometry exactly as described in the protocol detailed in section 3.4.1 in Hesketh et al. (2017).

### Mass spectrometry data acquisition

Both data-dependent acquisition (DDA) and data-independent acquisition (DIA) were performed. For DDA by liquid chromatography–tandem mass spectrometry (LC-MS/MS), affinity-purified and digested peptides were analyzed using a nano-HPLC (high-performance liquid chromatography) coupled to mass spectrometry. A quarter of the sample was used. Nano-spray emitters were generated from fused silica capillary tubing, with 100 µm internal diameter, 365 µm outer diameter and 5–8 µm tip opening, using a laser puller (Sutter Instrument Co., model P-2000; parameters set as heat=280, FIL=0, VEL=18, DEL=2000). Nano-spray emitters were packed with C18 reversed-phase material (Reprosil-Pur 120 C18-AQ, 3 µm) resuspended in methanol using a pressure injection cell. The sample in 5% formic acid was directly loaded at 800 nl/min for 20 min onto a 100 µm×15 cm nano-spray emitter. Peptides were eluted from the column with an acetonitrile gradient generated by an Eksigent ekspert™ nanoLC 425 and analyzed on a TripleTOF™ 6600 instrument (AB SCIEX, Concord, ON, Canada). The gradient was delivered at 400 nl/min from 2% acetonitrile with 0.1% formic acid to 35% acetonitrile with 0.1% formic acid using a linear gradient of 90 min. This was followed by a 15 min wash with 80% acetonitrile with 0.1% formic acid, and equilibration for another 15 min to 2% acetonitrile with 0.1% formic acid. The total DDA protocol was 135 min. The first DDA scan had an accumulation time of 250 ms within a mass range of 400–1800 Da. This was followed by 10 MS/MS scans of the top 10 peptides identified in the first DDA scan, with accumulation time of 100 ms for each MS/MS scan. Each candidate ion was required to have a charge state of 2–5 and a minimum threshold of 300 counts per second, isolated using a window of 50 mDa. Previously analyzed candidate ions were dynamically excluded for 7 s.

For DIA by LC–MS/MS, affinity-purified and digested peptides were analyzed using a nano-HPLC coupled to mass spectrometry. A quarter of the sample was used. Nano-spray emitters were generated from fused silica capillary tubing, with 100 µm internal diameter, 365 µm outer diameter and 5–8 µm tip opening, using a laser puller (Sutter Instrument Co., model P-2000; parameters set as heat=280, FIL=0, VEL=18, DEL=2000). Nano-spray emitters were packed with C18 reversed-phase material (Reprosil-Pur 120 C18-AQ, 3 µm) resuspended in methanol using a pressure injection cell. The sample in 5% formic acid was directly loaded at 800 nl/min for 20 min onto a 100 µm×15 cm nano-spray emitter. Peptides were eluted from the column with an acetonitrile gradient generated by an Eksigent ekspert™ nanoLC 425 and analyzed on a TripleTOF™ 6600 instrument (AB SCIEX, Concord, ON, Canada). The gradient was delivered at 400 nl/min from 2% acetonitrile with 0.1% formic acid to 35% acetonitrile with 0.1% formic acid using a linear gradient of 90 min. This was followed by a 15 min wash with 80% acetonitrile with 0.1% formic acid, and equilibration for another 15 min to 2% acetonitrile with 0.1% formic acid. The total DIA protocol was 135 min. The first DIA scan had an accumulation time of 250 ms within a mass range of 400–1800 Da. This was followed by 54 MS/MS scans with differing mass windows, with an accumulation time of 65 ms per scan.

### Mass spectrometry data analysis

Mass spectrometry data generated were stored, searched and analyzed using the ProHits laboratory information management system (LIMS) platform (Liu et al., 2016). Within ProHits, WIFF files were converted to an MGF format using the WIFF2MGF converter and to an mzML format using ProteoWizard (V3.0.10702) and the AB SCIEX MS Data Converter (V1.3 beta).

DDA acquisition data was searched using Mascot (V2.3.02; Perkins et al., 1999) and Comet (V2016.01 rev.2; Eng et al., 2013).The spectra were searched with the human and adenovirus sequences in the RefSeq database (version 57, January 30th, 2013) acquired from NCBI, supplemented with ‘common contaminants’ sequences from the Max Planck Institute (http://www.coxdocs.org/doku.php?id=maxquant:start_downloads.htm) and the Global Proteome Machine (GPM; https://www.thegpm.org/crap/), forward and reverse sequences (labelled ‘gi|9999’ or ‘DECOY’), sequence tags (BirA, GST26, mCherry and GFP) and streptavidin for a total of 72,481 entries. Database parameters were set to search for tryptic cleavages, allowing up to two missed cleavages sites per peptide with a mass tolerance of 35 ppm for precursors with charges of 2+ to 4+ and a tolerance of 0.15 amu for fragment ions. Variable modifications were selected for deamidated asparagine and glutamine, and oxidized methionine. Results from each search engine were analyzed through TPP (the Trans-Proteomic Pipeline, v.4.7 POLAR VORTEX rev 1) via the iProphet pipeline (Shteynberg et al., 2011).

DIA acquisition data was searched using MS-GFDB (Wang et al., 2015). The spectra were searched with the human and adenovirus sequences in the RefSeq database (version 57, January 30th, 2013) acquired from NCBI, supplemented with ‘common contaminants’ sequences from the Max Planck Institute the GPM (as above), for a total of 36,361 entries. Database parameters were set to search for tryptic cleavages, with a mass tolerance of 50 ppm for precursors with charges of 2+ to 4+ and a peptide length of 8–30 amino acids. Oxidized methionine was set as the variable modification. DDA files were used to generate a spectral library for the SWATH files.

### SAINT analysis

The SAINT analysis tool is used to identify high-confidence protein interactors versus control samples (Teo et al., 2014). SAINTexpress version 3.6.1 was used for DDA and version 3.6.3 was used for MSPLIT–DIA analysis. SAINT analysis was performed using two biological replicates per bait for both DDA and MSPLIT–DIA. Six negative control experiments with miniTurbo–3×FLAG alone samples were conducted for proximity-dependent biotinylation assays; two asynchronous replicates, two in G1/S and two in mitosis. SAINT probabilities were calculated independently for each sample, averaged (AvgP) across biological replicates and reported as the final SAINT score. Prior to applying SAINT, proteins were filtered with iProphet score≥0.95 and unique peptides≥2 for DDA and unique peptides≥2 for MSPLIT–DIA. Proteins with a BFDR (Bayesian FDR)≤0.01 were considered high-confidence protein interactors. Heat maps were generated from SAINT output via ProHits-viz (Knight et al., 2015).

### Filtered prey dataset

For each prey, the averaged spectral counts obtained with miniTurbo alone (two replicates for each of the three conditions) were subtracted from averaged spectral counts with miniTurbo–3×FLAG–CNAα (wild type or mutant) for each condition (background subtracted spectral counts; Table S1). The final dataset of 41 proteins resulted from 38 preys from DDA analysis and 3 additional preys from MSPLIT–DIA analysis that were significantly biotinylated by miniTurbo–3×FLAG–CNAα_WT_ (unique peptides≥2, BFDR≤0.01) in at least one condition. PxIxIT docking-dependent proteins displayed a log_2_ spectral count ratio (miniTurbo–3×FLAG–CNAα_WT_/miniTurbo–3×FLAG–CNAα_NIRmut_) greater than or equal to 0.5 for at least one condition. For preys with a miniTurbo–3×FLAG–CNAα_NIRmut_ spectral count=0, values were converted to 0.5 to calculate spectral count ratios.

### GST-tagged PxIxIT motif peptide purification

Oligos encoding 16mer peptides with PxIxIT motifs in the center were fused to GST in a pGEX-4T-3 vector (Cytiva, 28954552) and expressed in BL21 (DE3) chemically competent *E. coli* (Sigma-Aldrich, CMC0014). Bacteria were grown at 37°C until mid-log phase, and expression was induced with 1 mM isopropyl-β-D-thiogalactopyranoside (IPTG; P212121, GB-I0920) addition for 2 h. Bacteria were lysed with CelLytic B Cell Lysis Reagent (Millipore-Sigma, B7435) according to the manufacturer's instructions. Cell lysates expressing GST-tagged peptides were incubated with Glutathione Sepharose 4B beads (Cytiva, 17-0756-01) at 4°C for 2–4 h, and the beads were then isolated and eluted through a Bio-Spin Chromatography Column (Bio-Rad, #7326008) with elution buffer (50 mM Tris-HCl pH 8, 300 mM NaCl, 0.1% NP-40, 5 mM DTT and 40 mM glutathione; NaOH added to adjust to pH 8). The eluates were allowed to dialyze at 4°C overnight in dialysis buffer (100 mM Tris-HCl pH 8, 150 mM NaCl, 1 mM β-mercaptoethanol) to remove residual glutathione. Purified peptides were stored in 10% glycerol at −80°C.

### 6×His-tagged CN and maltose binding protein purification

6×His-tagged human CNAα (truncated at residue 392), either WT or with the ^330^NIR^332^ to ^330^AAA^332^ PxIxIT-docking mutation, was expressed in tandem with the calcineurin B subunit (PPP3R1, amino acids 1–170) in a p11 vector (PSI:Biology-Materials Repository) in BL21 (DE3) chemically competent *E. coli*. Similarly, 6×His-tagged WT maltose binding protein (MBP) was expressed in a p11 vector in BL21 (DE3) chemically competent *E. coli*. Bacteria were grown at 37°C until mid-log phase, and expression was induced with 1 mM IPTG at 16°C for 18 h. Cells were pelleted, washed and frozen at −80°C for at least 12 h. Thawed cell pellets were resuspended in lysis buffer [50 mM Tris-HCl pH 7.5, 150 mM NaCl, 0.1% Tween-20, 1 mM β-mercaptoethanol and Halt protease inhibitors (Thermo Fisher Scientific, 78430)] and lysed by sonication using four 1-min pulses at 40% output. Extracts were clarified using two rounds of centrifugation (20,000 ***g***, 20 min, 4°C) and then bound to Ni-NTA agarose beads (Invitrogen, R901-15) in lysis buffer containing 5 mM imidazole for 2–4 h at 4°C. Bound beads were loaded onto a Bio Spin Chromatography Column and washed with lysis buffer containing 20 mM imidazole (Sigma-Aldrich, I0250) and eluted with lysis buffer containing 300 mM imidazole, pH 7.5. Purified proteins were dialyzed in buffer (50 mM Tris-HCl pH 7.5, 150 mM NaCl, 1 mM β-mercaptoethanol) and stored in 10% glycerol at −80°C.

### *In vitro* GST-tagged peptide binding

1–2 μg of purified 6×His-tagged CN, or 6×His–MBP as a negative control, prepared as described above, was first bound to 10 μl magnetic Dynabeads His-Tag Isolation and Pulldown beads (Thermo Fisher Scientific, 10104D) in 490 μl base buffer (50 mM Tris-HCl pH 7.5,150 mM NaCl, 0.1% Tween 20, 1 mM β-mercaptoethanol, Halt protease inhibitors), supplemented with 15 mM imidazole and 0.5 mg/ml BSA for 1.5 h at 4°C. 7–10 μg of appropriate purified GST-tagged peptides was then added to the binding reaction and incubated further for 2 h at 4°C. 3% of the total reaction mix was removed as ‘input’ prior to the incubation, boiled in Laemmli sample buffer and stored at −20°C. The beads were washed in base buffer containing 20 mM imidazole, and bound proteins were eluted by boiling in Laemmli sample buffer for 5 min, followed by SDS–PAGE and immunoblotting. GST-tagged peptides co-purifying with 6×His-tagged proteins were normalized to their respective input and the amount of His-tagged protein pulled down. Co-purification with CN was reported relative to that of the peptide with the known PxIxIT motif from NFATC1: PALESPRIEITSCLGL. POC5 peptides used were: POC5 PDVRIS, KGELVPDVRISTIHDI; and POC5 ADARAA Mut, KGELVADARAATIHDI. Statistical significance was determined by an unpaired, two-tailed Student's *t*-test, using GraphPad Prism 9. *In vitro* GST pulldown experiments were performed in three biological replicates.

### Calcineurin and POC5 co-immunoprecipitation assays

HEK293T cells transfected with plasmids expressing 6×myc-tagged POC5 (WT or the ^35^ADARAA^40^ mutant, POC5_ADARAA_) and GFP-tagged FLAG or GFP–CNAα_WT_ were washed with PBS and harvested. Human POC5 sequences (amino acids 1–550; UniProt ID Q8NA72-3), either with WT residues 35–40 (PDVRIS) or with residues 35–40 mutated to ADARAA, were inserted into plasmid pLenti-6×myc-N1 (a gift from Christian Hoerner, Stanford University, CA, USA). GFP was inserted into pDEST pcDNA5-FRT-3×FLAG (Lambert et al, 2013). GFP fused to CNAα amino acid residues 1–521 was inserted into pcDNA5 FRT TO (Invitrogen, V652020). Cell pellets were snap-frozen in liquid nitrogen and stored at −80°C until use. Thawed cell pellets were lysed with lysis buffer (50 mM Tris-HCl pH 7.5, 150 mM NaCl, 1% NP-40) supplemented with Halt Protease and Phosphatase Inhibitor Cocktail (Thermo Fisher Scientific, 78440) and subjected to fine needle aspiration through a sterile 27.5G needle. Cell lysates were clarified by centrifugation at 16,000 ***g*** for 20 min in 4°C, and protein concentrations were determined using the Pierce BCA Protein Assay Kit, according to the manufacturer's instructions. 1 mg of protein from each lysate was added to 20 μl of pre-washed anti-c-myc magnetic beads (Med Chem Express, HY-K0206) and the volume of each reaction was equalized to 500 μl with binding buffer (50 mM Tris-HCl pH 7.5, 150 mM NaCl, 0.5% NP-40, Halt Protease and Phosphatase Inhibitor Cocktail). 2.5% of the total reaction mix was removed as ‘input’ prior to the incubation, boiled in Laemmli sample buffer, and stored at −20°C. The reactions were then rotated gently at 4°C overnight. Beads were washed four times with wash buffer (0.5% Triton X-100, Halt Protease and Phosphatase Inhibitor Cocktail in PBS) and boiled in 2× Laemmli sample buffer for 5 min. 50% of ‘input’ and ‘immunoprecipitated' fractions were resolved by SDS–PAGE and immunoblotted. Four biological replicates of this experiment were performed. GFP-tagged proteins co-immunoprecipitating with 6×myc-tagged POC5 were normalized to their respective input and then over the amount of 6×myc-POC5 bound to the beads. Statistical significance was determined with a ratio-paired, two-tailed Student's *t*-test, using GraphPad Prism 9.

### Immunoprecipitation and *in vitro* dephosphorylation of POC5

HeLa cells were transfected with the plasmid expressing 6×myc–POC5_WT_ and divided between two plates: one where cells were treated with DMSO and another where cells were treated with 100 ng/ml nocodazole (Cell Signaling Technology, 2190S) for 18 h at 37°C. DMSO or nocodazole were washed out with PBS, and cells were incubated with fresh, drug-free medium for 1 h at 37°C. Cells were harvested, pelleted, frozen in liquid nitrogen and stored at −80°C until use. Thawed cell pellets were lysed with lysis buffer (50 mM Tris-HCl pH 7.5, 150 mM NaCl, 1% NP-40) supplemented with Halt Protease and Phosphatase Inhibitor Cocktail and subjected to fine needle aspiration through a sterile 27.5G needle. Cell lysates were clarified by centrifugation at 16,000 ***g*** for 20 min at 4°C, and protein concentrations were determined using the Pierce BCA Protein Assay Kit, according to the manufacturer's instructions. 500 μg of protein from each lysate was added to 20 μl of pre-washed anti-c-myc magnetic beads (Med Chem Express, HY-K0206), and the volume of each reaction was equalized to 500 μl with binding buffer (50 mM Tris-HCl pH 7.5, 150 mM NaCl, 0.5% NP-40, Halt Protease and Phosphatase Inhibitor Cocktail). 2.5% of the total reaction mix was removed as ‘input’ prior to the 2 h incubation, boiled in 2× Laemmli sample buffer and stored at 4°C. The bead binding mixtures were then rotated gently at 4°C for 2 h. Beads were washed twice with wash buffer (0.5% Triton X-100, Halt Protease and Phosphatase Inhibitor Cocktail in PBS) and then washed twice with either λ dephosphorylation buffer [1 mM MnCl_2,_ 1× PMP buffer (New England Biolabs, P0753) and Halt protease inhibitors] or CN dephosphorylation buffer (50 mM Tris-HCl pH 8, 100 mM NaCl, 6 mM MgCl_2,_ 1 mM CaCl_2,_ 1 mM DTT, Halt protease inhibitors). Finally, the beads were incubated in λ dephosphorylation buffer or CN dephosphorylation buffer, with or without phosphatase addition [0.25 μl of λ phosphatase (New England Biolabs, P0753) in 50 μl reaction volume, or 200 nM purified constitutively active truncated 6×His–CNAα_WT_:CNB] and with or without phosphatase inhibitor addition (Halt Protease and Phosphatase Inhibitor Cocktail and 5 mM EDTA), as required. Dephosphorylation was allowed to occur for 45 min at 30°C under constant shaking. Reactions were stopped with 2× Laemmli buffer, and samples were then boiled for 5 min. Proteins were analyzed on 6% acrylamide SDS–PAGE gels followed by immunoblotting. *In vitro* dephosphorylation experiments were performed in three biological replicates.

### Cytosol extraction and immunofluorescence microscopy

HeLa, IMCD3 or hTERT-RPE1 cells were grown on poly-L-lysine (Sigma-Aldrich, P4707) pre-treated 12 mm, #1.5H glass coverslips (ThorLabs). If cytosol extraction was not required, cells were directly fixed in ice-cold methanol at −20°C for 15 min. For cytosol-extracted hTERT-RPE1 cells, the coverslips were first washed with warm 0.02% digitonin in PBS solution and rocked gently for 5 min at room temperature, followed by one wash with PBS and methanol fixation at −20°C for 15 min. Following methanol fixation, coverslips were washed thrice with PBS and then placed in a dark humid chamber and treated with blocking buffer (0.2 M glycine, 0.1% Triton X-100, 2.5% FBS in PBS) for 30 min. Coverslips were incubated with primary antibodies diluted in blocking buffer for 1 h, washed multiple times with PBS, followed by incubation with secondary antibodies for 45 min, multiple washes with PBS and a 15-min incubation with 5 μg/ml DAPI at room temperature. Coverslips were washed again and mounted on glass slides using ProLong Diamond Antifade Mountant (Thermo Fisher Scientific, P36965).

Imaging was performed at the Stanford University Cell Sciences Imaging Facility, using an Inverted Zeiss LSM 880 confocal laser scanning microscope (CSIF; RRID:SCR_017787) with either a 1.3 NA 40× EC Plan Neo oil immersion objective or a 1.4 NA 63× Plan Apo oil immersion objective. Lasers used were diode 405 nm, 0.2–0.8 mW; HeNe 594 nm, 2 mW; and Ar 488 nm, 25 mW. Images were acquired at constant exposure settings within experiments using the Zen Black software (Carl Zeiss). ImageJ was used for image analysis, line intensity plots and quantification of fluorescence intensities.

### Ultrastructure expansion microscopy

hTERT-RPE1 cells were grown on 12 mm, #1.5H glass coverslips and treated either with DMSO (control) or 2.5 μM FK506 (LC laboratories, F4900) for 48 h. Coverslips were fixed in ice-cold methanol at −20°C for 10 min and washed with PBS. Following fixation, U-ExM was performed as previously described (Gambarotto et al., 2019). Briefly, coverslips were incubated overnight in an acrylamide–formaldehyde solution (AA/FA; 0.7% formaldehyde, 1% acrylamide in PBS) at 37°C. Gelation was allowed to proceed in monomer solution (19% sodium acrylate, 10% acrylamide, 0.1% bis-acrylamide, 0.5% ammonium persulfate-APS, 0.5% TEMED), and the coverslips were discarded. Gels were heated in denaturation buffer (200 mM SDS, 200 mM NaCl, 50 mM Tris-HCl pH 9) at 95°C for 1 h. After denaturation buffer was removed, gels were washed with multiple water rinses and allowed to expand in water at room temperature overnight. Small circles of each expanded gel (∼5 mm in diameter) were excised and incubated with primary antibodies diluted in PBSBT buffer (3% BSA, 0.1% Triton X-100 in PBS) on a nutator at 4°C overnight. The next day, gels were washed thrice with PBSBT buffer and incubated with secondary antibodies and 5 μg/ml DAPI diluted in PBSBT, protected from light, on a nutator at 4°C overnight. Immunostained gels were washed once with PBS and thrice with water, and placed in a glass-bottomed, poly-L-lysine-treated 35 mm plate for imaging. All U-ExM images were acquired as single planes or *z*-stacks collected at 0.27-μm intervals using a confocal Zeiss Axio Observer microscope (Carl Zeiss) with a PlanApoChromat 1.4 NA 63× oil immersion objective, a Yokogawa CSU-W1 head and a Photometrics Prime BSI express CMOS camera. Slidebook software (Intelligent Imaging Innovations, 3i) was used to control the microscope system. Deconvolution was performed with Microvolution (Cupertino, CA, USA) using a calculated point spread function (PSF) for 10 iterations. ImageJ was used for image analysis and quantification of fluorescence intensities.

### Sucrose fractionation for nuclear/centrosomal fractions

HeLa cells were washed once in PBS buffer then centrifuged for 3 min at 300 ***g*** at room temperature, and the pellet was resuspended with lysis buffer [10 mM Tris-HCl, pH 7.5, 10 mM NaCl, 3 mM MgCl_2_, 1% NP-40, 10% sucrose and cOmplete protease inhibitors (Millipore Sigma, 4693159001)] at 1 ml per 1×10^7^ cells. Lysed cells were centrifuged at 100 ***g*** at 4°C for 5 min. A small fraction of the supernatant was flash-frozen and stored in −80°C as the cytosolic fraction, and the rest was discarded. The pellet containing the nuclei and centrosomes was incubated at room temperature for 30 min in the same volume of digestion buffer (10 mM K-PIPES pH 6.8, 50 mM NaCl, 3 mM MgCl_2_, 1 mM EGTA, 10% sucrose, 0.1 mg/ml DNAse I, 0.1 mg/ml RNAse A) as the volume used for lysis buffer. (NH_4_)_2_SO_4_ and NaCl solutions were then added at final concentrations of 0.25 M and 1 M, respectively. The mixture was centrifuged at 15,000 ***g*** for 15 min over a 1 ml cushion made of 60% sucrose in digestion buffer. After centrifugation, 2 ml of material (1 ml of supernatant and 1 ml of cushion) was collected at the 10–60% sucrose interphase and contents were vortexed, flash-frozen and stored at −80°C.

### Anti-CNB antibody blocking

60 μg of BSA protein (Sigma-Aldrich, A3294) and 60 μg purified constitutively active 6×His–CNAα_WT_:CNB were resolved by SDS–PAGE and transferred to a nitrocellulose membrane (Bio-Rad, 162-0112). The membrane was stained with Ponceau S solution (Sigma-Aldrich, P7170) for 15 min and rinsed twice with distilled water to visualize the bands of interest. The membrane was excised around the bands corresponding to BSA and CNB, and the membrane strips were first washed with water to remove excess Ponceau, and then blocked with 5% milk in TBST buffer (Tris-buffered saline with 0.1% Tween-20) for 1 h at room temperature. The strips were then rinsed twice in TBST buffer, and twice in PBSBT buffer. Finally, each strip was incubated with 99 μl PBSBT and 1 μl mouse anti-CNB antibody (Sigma-Aldrich, C0581) on a nutator for 5 h at room temperature. The membrane strips were then discarded, and the antibody mixture was added onto coverslips for immunofluorescence staining as described above.

### Measurement of centriole length and percentage coverage of POC5 and γ-tubulin

All measurements were performed with ImageJ using single *z*-plane images obtained by U-ExM of hTERT-RPE1 centrioles after 48 h treatment with DMSO or 2.5 μM FK506. Overall centriole length was quantified using acetylated tubulin staining, and coverage length was determined by anti-POC5 or anti-γ-tubulin staining, exactly as has been previously described for GCP4 coverage (Schweizer et al., 2021). Centrioles included in these measurements displayed a longitudinal axis parallel to the *x*–*y* plane and were primarily parental centrioles in G1 phase or S phase. Number of centrioles analyzed for Fig. 3H,I: DMSO, *n*=60; FK506, *n*=58. Number of centrioles analyzed for Fig. S3J: DMSO, *n*=46; FK506, *n*=51. For Fig. 3J, centrioles from Fig. 3H,I that displayed a visible lumen by acetylated tubulin staining were measured.

### Measurement of ciliation and cilia length

To measure changes during active ciliogenesis, IMCD3 cells were grown to 90% confluency on a 10 cm tissue culture plate containing two 12 mm, #1.5H glass coverslips. The coverslips were removed and fixed in 4% PFA for 10 min in room temperature and then prepared for immunofluorescence. The remaining cells on the plate were trypsinized and split into two fully confluent 3.5 cm tissue culture plates with glass coverslips. One plate contained medium supplemented with DMSO and the other with 2.5 μM FK506. 24 h after drug treatment, two coverslips from the DMSO-treated plate and two from the FK506-treated plate were removed, fixed and stained for immunofluorescence. The same procedure was performed again 48 h after drug treatment began. This experiment was performed in triplicate. Number of cilia measured to determine cilia length in Fig. 4C: 0 h, *n*=331; 24 h DMSO, *n*=556; 24 h FK506, *n*=256; 48 h DMSO, *n*=931; 48 h FK506, *n*=429.

To measure changes in the maintenance of existing cilia, IMCD3 cells were grown to confluency on 12 mm, #1.5H glass coverslips. Two days after reaching confluency, cells were treated with DMSO, 10 μM forskolin (Sigma-Aldrich, F3917), 2.5 μM FK506 or 2.5 μM cyclosporin A (Sigma-Aldrich, 30024) for 3 h at 37°C. Coverslips were fixed in 4% PFA for 10 min in room temperature and then prepared for immunofluorescence. Four biological replicates of this experiment were performed. Number of cilia measured to determine cilia length in Fig. 4E: DMSO, *n*=537; forskolin, *n*=514; cyclosporin A, *n*=553; FK506, *n*=638.

Immunofluorescence and image acquisition was performed as detailed in the ‘Cytosol extraction and immunofluorescence microscopy’ section above. ImageJ was used to count the number of cells (determined from DAPI-stained nuclei in a maximum projection of all *z*-planes) in each image. The CiliaQ plugin for ImageJ (https://github.com/hansenjn/CiliaQ; Hansen et al., 2021) with the CANNY 3D segmentation method was used to determine the number and lengths of Arl13b-stained cilia from *z*-slices collected at 0.5 μm intervals in the 488 nm channel. Using R Studio, cilia length data were accumulated and filtered so that only single-branched cilia with length over 1 μm were selected for further analysis.

Percent ciliation was determined by dividing the number of cilia with length>1 μm (Arl13b as a 3D vector in a *z*-stack) by the number of nuclei (DAPI in maximum projection images of a confocal *z*-stack), and multiplying the result by 100. Number of cells analyzed to determine percent ciliation in Fig. 4B: 0 h, *n*=1319; 24 h DMSO, *n*=2176; 24 h FK506, *n*=2468; 48 h DMSO, *n*=3710; 48 h FK506, *n*=4120. Number of cells analyzed to determine percent ciliation in Fig. 4F: >1200 cells per replicate per treatment.

### Antibodies

All antibodies used in this study were commercial antibodies, analyzed by immunofluorescence or immunoblotting and validated by the expected size and/or intracellular localization as described in each manufacturer's webpage. Primary antibodies used in immunoblotting: anti-6×His (1:3000; Takara Bio, 631212; RRID:AB_2721905), anti-β-actin (1:3000; LI-COR Biosciences, 926-42210; RRID:AB_1850027), anti-cyclin A2 (1:5000; ABclonal, A2891; RRID:AB_2764711), anti-FLAG M2 (1:5000; Sigma-Aldrich, F1804; RRID:AB_262044), anti-GFP (1:3000; Santa Cruz Biotechnology, sc-9996; RRID:AB_627695), anti-GST (1:3000; Cytiva, 27-4577-01; RRID:AB_771432), anti-myc-tag (1:3000; Cell Signaling Technology, 2278; RRID:AB_490778), anti-phospho-histone H3 Ser10 (1:500; Millipore, 06-570; RRID:AB_310177). Secondary antibodies used in immunoblotting: IRDye 680RD goat anti-mouse IgG (H+L) (1:15,000; LI-COR Biosciences, 926-68071; RRID:AB_10956166), IRDye 800CW donkey anti-goat IgG (H+L) (1:15,000; LI-COR Biosciences, 926-32214; RRID:AB_621846), IRDye 800CW goat anti-rabbit IgG (H+L) (1:15,000; LI-COR Biosciences, 926-32211; RRID:AB_621843). Primary antibodies used in immunofluorescence: anti-Arl13b (1:250; Proteintech, 17711-1-AP; RRID:AB_2060867), anti-calcineurin pan A (1:100; Millipore, 07-1491; RRID:AB_10562920), anti-centrin 2 (1:500; Millipore, 04-1624; RRID:AB_10563501), anti-centrin 3 (1:500; Novus, H00001070-M01; RRID:AB_537701); anti-CNB (1:100; Sigma-Aldrich, C0581; RRID:AB_258693), anti-gamma tubulin (1:500; Abcam, ab11316; RRID:AB_297920), anti-myc-tag (1:300; Cell Signaling Technology, 2278; RRID:AB_490778), anti-POC5 (1:100; Thermo Fisher Scientific, PA5-24308; RRID:AB_2541808), anti-polyglutamylation modification (1:500; AdipoGen, AG-20B-0020; RRID:AB_2490210), anti-tubulin, acetylated (1:500; Sigma-Aldrich, T6793; RRID:AB_477585). Secondary antibodies used in immunofluorescence: goat anti-rabbit IgG (H+L) Alexa Fluor 488 (1:1000; Thermo Fisher Scientific, A-11008; RRID:AB_143165), goat anti-mouse IgG1 Alexa Fluor 488 (1:1000; Thermo Fisher Scientific, A-21121; RRID:AB_2535764), goat anti-mouse IgG2b Alexa Fluor 568 (1:500; Thermo Fisher Scientific, A-21144; RRID:AB_2535780), goat anti-mouse IgG2a Alexa Fluor 568 (1:500; Thermo Fisher Scientific, A-21134; RRID:AB_2535773), goat anti-rabbit IgG (H+L) Alexa Fluor 568 (1:500; Thermo Fisher Scientific, A-11011; RRID:AB_143157), goat anti-mouse IgG (H+L) Alexa Fluor 594 (1:1000; Thermo Fisher Scientific, A-11032; RRID:AB_2534091), goat anti-rabbit IgG (H+L) Alexa Fluor 647 (1:500; Thermo Fisher Scientific, A-21245; RRID:AB_2535813), goat anti-mouse IgG2b Alexa Fluor 647 (1:500; Thermo Fisher Scientific, A-21242; RRID:AB_2535811).

### Statistics and study design

No statistical methods were used to determine sample size prior to experiments. Sample sizes for each experiment are explicitly stated in the article. The number of cells or cilia analyzed for quantifications were determined based on previous experience and/or on previously published similar experiments. No data were excluded from analyses, unless explicitly stated in the Materials and Methods. The majority of biochemical assays were repeated at least three times, and all attempts of replication were successful. No randomization was deemed necessary as all experiments were conducted with appropriate positive and negative controls. No blinding was deemed necessary due to clear effects observed and because all samples in biochemical, blotting and imaging experiments were analyzed in exactly the same manner. The type of significance testing conducted for each experiment is explicitly stated in the article. Statistical tests were conducted using GraphPad Prism 9. Statistical significance is represented in each figure as follows: ns, not significant; *P<0.05; **P<0.01; ***P<0.001.

## Supplementary Material

10.1242/joces.260353_sup1 Supplementary information
