# Supplementary material for: Calcineurin associates with centrosomes and regulates cilia length maintenance
Source: J Cell Sci. 2023 Apr 24;136(8):jcs260353. doi: 10.1242/jcs.260353 (PMC10163345; doi:10.1242/jcs.260353)
Supplement: Supplementary information [file joces-136-260353-s1.pdf]

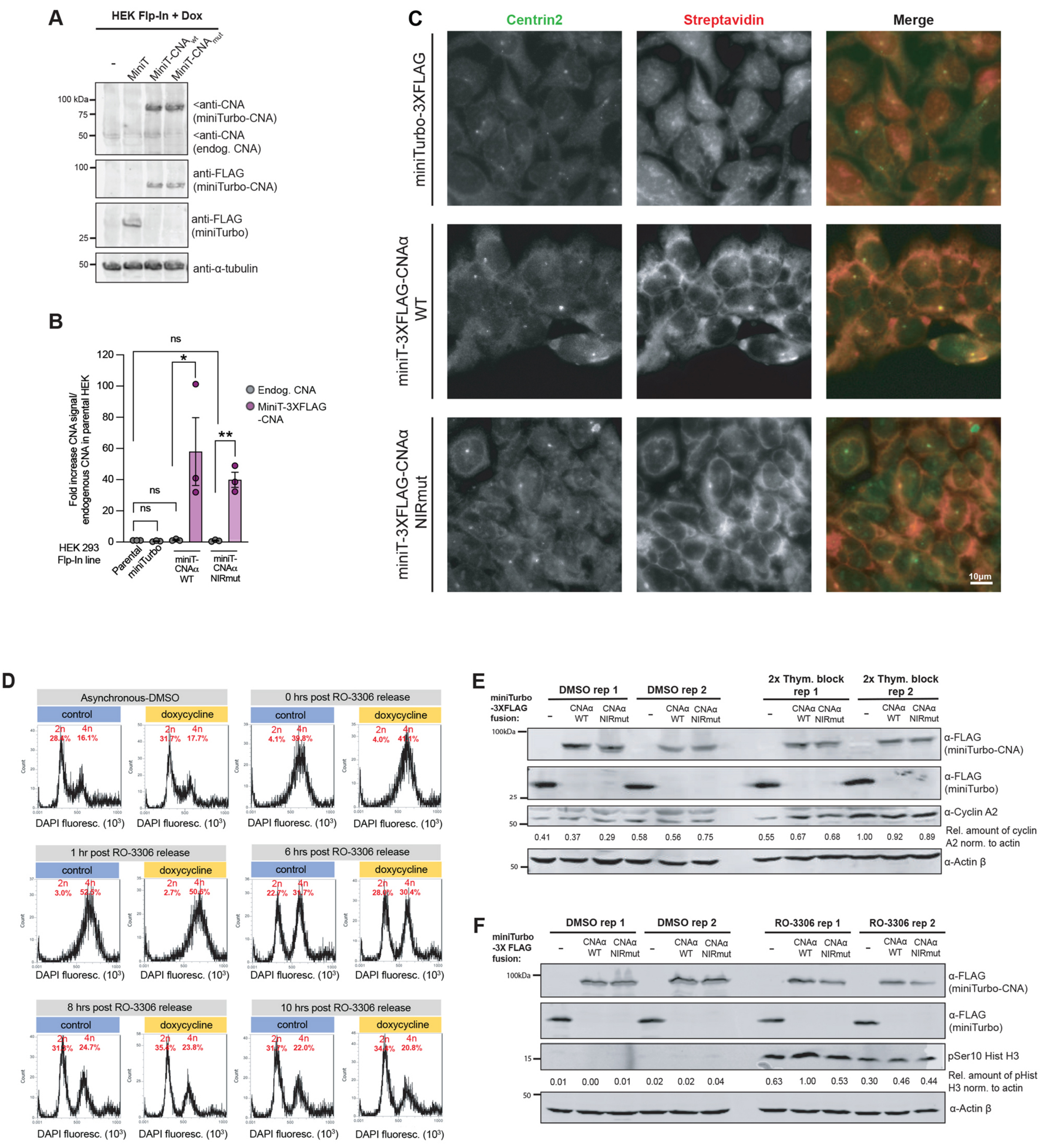
A
HEK Flp-In + Dox
<anti-CNA (miniTurbo-CNA)
<anti-CNA (endog. CNA)
anti-FLAG (miniTurbo-CNA)
anti-FLAG (miniTurbo)
anti-α-tubulin
B
Fold increase CNA signal/ endogenous CNA in parental HEK
Endog. CNA
MiniT-3XFLAG -CNA
HEK 293 Flp-In line
C
Centrin2
Streptavidin
Merge
miniTurbo-3XFLAG
miniT-3XFLAG-CNAα WT
miniT-3XFLAG-CNAα NIRmut
10µm
D
Asynchronous-DMSO
0 hrs post RO-3306 release
1 hr post RO-3306 release
6 hrs post RO-3306 release
8 hrs post RO-3306 release
10 hrs post RO-3306 release
control
doxycycline
E
DMSO rep 1
DMSO rep 2
2x Thym. block rep 1
2x Thym. block rep 2
α-FLAG (miniTurbo-CNA)
α-FLAG (miniTurbo)
α-Cyclin A2
Rel. amount of cyclin A2 norm. to actin
α-Actin β
F
DMSO rep 1
DMSO rep 2
RO-3306 rep 1
RO-3306 rep 2
α-FLAG (miniTurbo-CNA)
α-FLAG (miniTurbo)
pSer10 Hist H3
Rel. amount of pHist H3 norm. to actin
α-Actin β

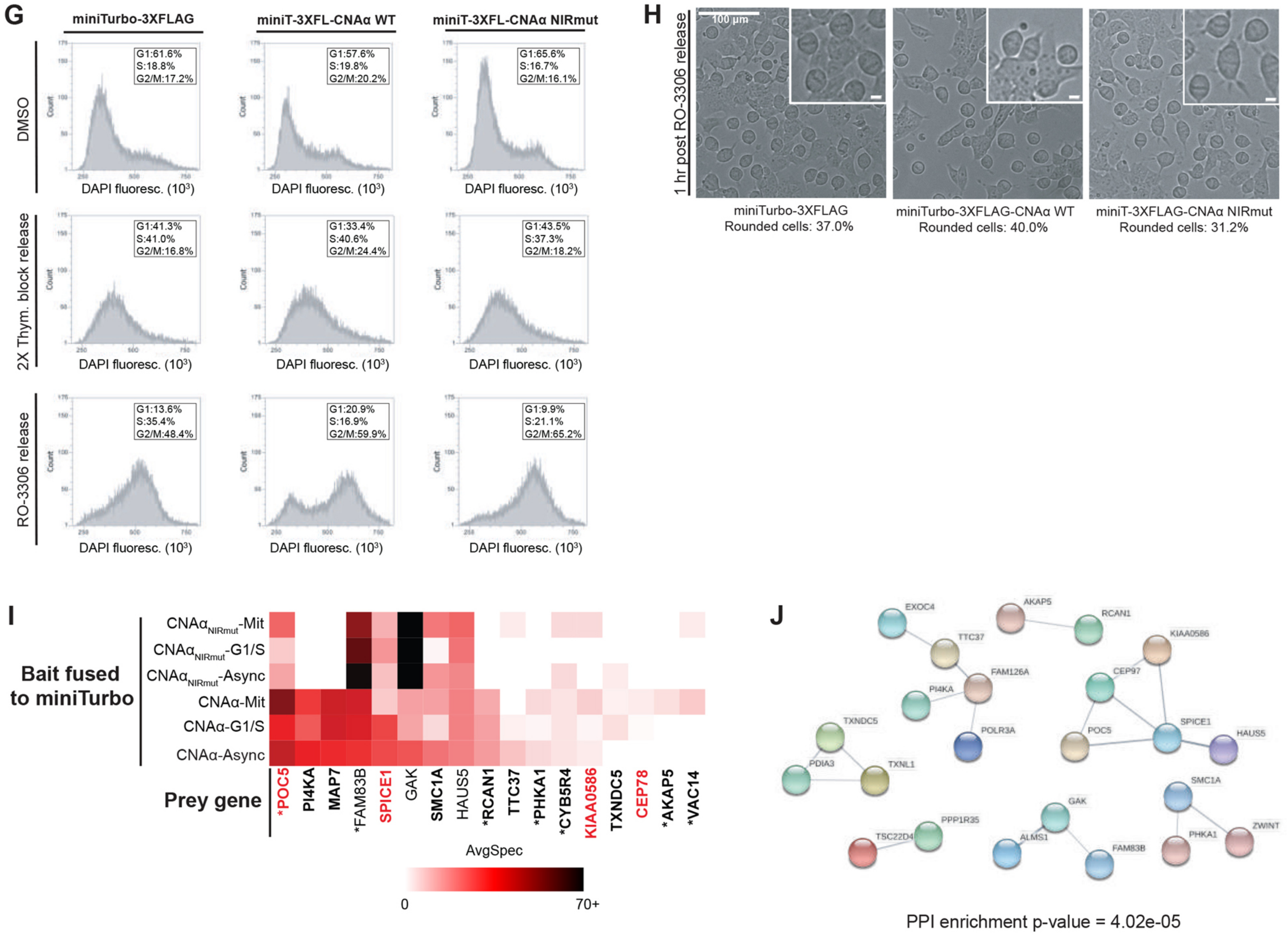

Fig. S1 (Related to Figure 1). Proximity-labeled cells showed equal levels of miniTurbo bait expression, proper cell cycle synchronization and resulted in preys known to associate with one another.

A. Endogenous CNA expression compared to miniTurbo-fused CNA expression in HEK293 Flp-In T-Rex cell lines. Immunoblot with lysates from asynchronous parental cells or cells induced to express miniTurbo fusions. Dox, 1 µg/mL doxycycline for 48 hours.

B. Quantification of CNA expression from immunoblot shown in A. Data represent anti-CNA signal normalized to α-tubulin loading control, relative to endogenous CNA expression in parental HEK293 Flp-In T-Rex cells. Bars represent the mean ± SEM (n=3 independent experiments). n.s., not significant, *$p < 0.05$, **$p < 0.01$, ***$p < 0.001$. p-values determined by ratio-paired, two-tailed t-test.

C. Biotinylation in asynchronous miniTurbo cell lines. HEK293 Flp-In T-Rex cells expressing miniTurbo, miniTurbo-CNAα_{WT} or miniTurbo-CNAα_{NIRmut} were labelled with biotin and imaged with fluorophore-conjugated streptavidin. Images obtained from maximum projections of z-stacks. Scale bar, 10 µm.

Journal of Cell Science • Supplementary information

D. Induction of miniTurbo-CNAα overexpression does not disrupt cell cycle progression. HEK293 Flp-In T-Rex with inducible miniTurbo-CNAα_{WT} expression were either treated with 0 µg/mL doxycycline (control-blue boxes) or 1 µg/mL doxycycline (doxycycline-yellow boxes) for 48 hours. On the second day of doxycycline induction, cells were also treated with DMSO (asynchronous) or 9 µM RO-3306 for 20 hours and then released from arrest. At various time points post RO-3306 washout, cells were fixed, stained with 20 µg/mL DAPI and analyzed by flow cytometry. Cell cycle profiles post-release were similar between the control and doxycycline groups.

E. Protein expression in asynchronous and G1/S miniTurbo samples. HEK293 Flp-In T-Rex cells with induced expression of miniTurbo fusions were synchronized and labeled with biotin. Immunoblot contains samples from two independent experimental replicates. Anti-FLAG staining was used to ensure expression of the miniTurbo baits, anti-cyclin A2 as a cell cycle marker and anti-actin beta as a loading control. Rep, replicate. Rel. amount of cyclin A2 norm. to actin: relative ratio of cyclin A2 band signal normalized by the corresponding actin band signal.

F. Protein expression in asynchronous and mitotic miniTurbo samples. HEK293 Flp-In T-Rex cells with induced expression of miniTurbo fusions were synchronized and labeled with biotin. Immunoblot contains samples from two independent experimental replicates. Anti-FLAG staining was used to ensure expression of the miniTurbo baits, anti-Ser10 phosphorylated histone H3 as a mitotic marker and anti-actin beta as a loading control. Rep, replicate. Rel. amount of pHistone H3 norm. to actin: relative ratio of phosphorylated histone H3 band signal normalized by the corresponding actin band signal.

G. Cell cycle profiles of synchronized miniTurbo HEK293 Flp-In T-Rex cells prior to mass spectrometry analysis. Profiles shown are from one of the two independent replicates of the proximity labeling experiment and were obtained by flow cytometry using DAPI staining.

H. Brightfield microscopy images showing miniTurbo cell samples synchronized in mitosis 1 hour after RO-3306 washout. Insets show that cells are rounded up and their chromosomes are aligned on the metaphase plate, indicating that they are mitotic. Scale bar, 100 µm, inset scale bar, 10 µm.

Journal of Cell Science • Supplementary information

I. Heat map of average spectral counts for preys labeled by miniTurbo-CNA_{WT} or CNA_{NIRmut} in asynchronous, G1/S and mitotic cells. Only preys labeled by CNA_{WT} with unique peptides ≥ 2 and bayesian false discovery rate (BFDR) ≤ 0.01 in at least one treatment are included. Spectral counts were obtained via data-independent mixture-spectrum partitioning using libraries of identified tandem mass spectra (DIA/mSPLIT). Bold: preys with PxIxIT-dependent biotinylation (Log_2 CNA_{WT}/CNA_{NIRmut} ≥ 0.5). Red: proteins annotated with the Gene Ontology (GO) term “centrosome”. Asterisks: proteins with predicted CN-dependent SLiMs (PxIxIT or LxVP) identified *in silico* (Wigington et al., 2020). AvgSpec, average spectral counts. Exact spectral counts can be found under “mSPLIT filtered dataset” on Table S1.

J. STRING database v11.5 (string-db.org) network of protein-protein interactions between CN-proximal proteins identified in this study (38 from DDA analysis and 3 additional from mSPLIT analysis). Proteins without any known interactors within the network were omitted. Line intensity is proportional to the confidence of the interaction. PPI enrichment p-value: 4.02e-05. Number of edges: 19. Expected number of edges: 6.

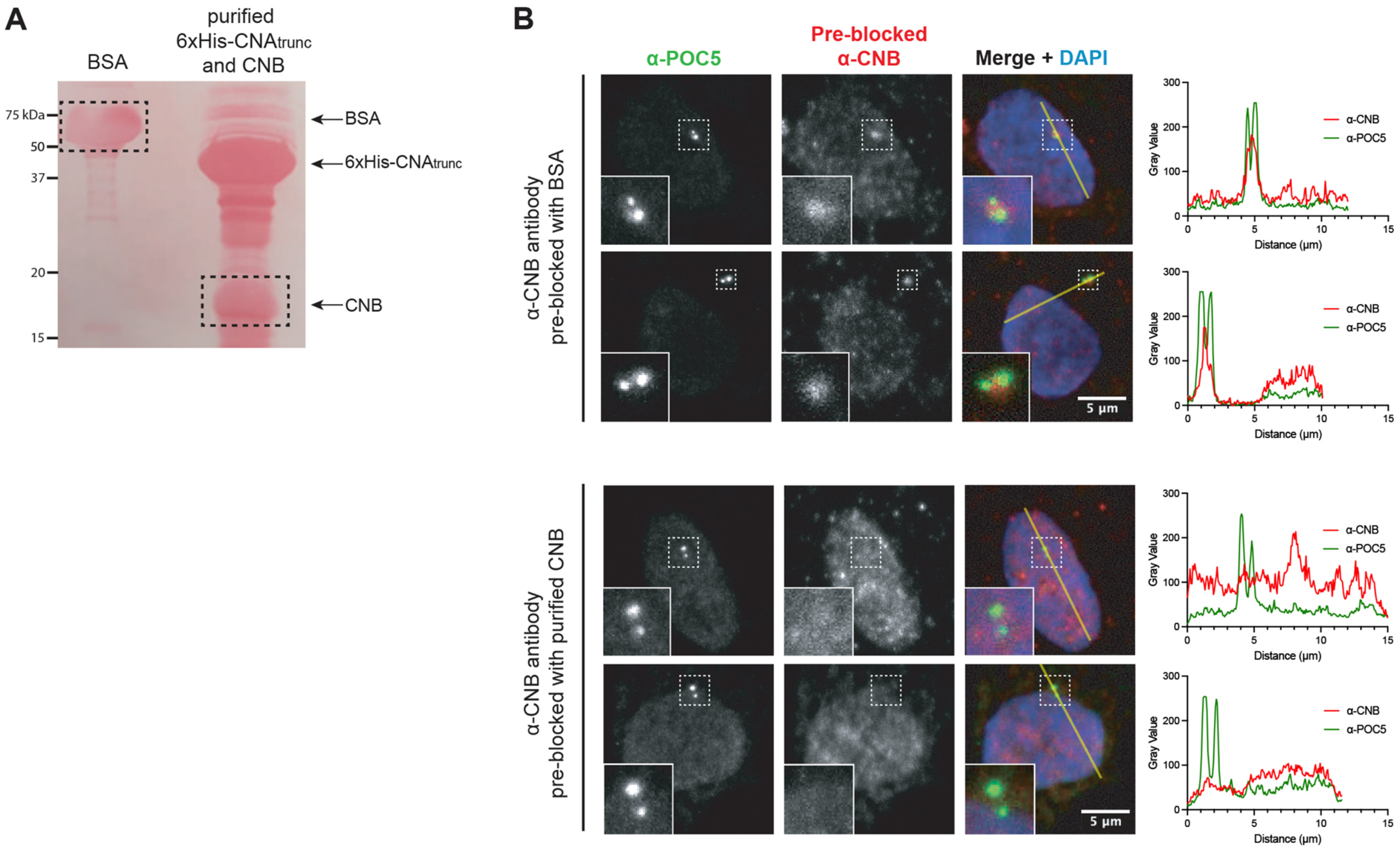

Fig. S2. (Related to Figure 2) CNB antibody blocking demonstrates that centrosomal localization of CNB is highly specific.

A. Nitrocellulose membrane stained with Ponceau S, showing analysis of purified BSA and purified truncated CNA and CNB by SDS-PAGE. Dotted lines indicate pieces of the membrane that were excised and incubated with anti-CNB antibody for antibody blocking in panel B.

B. Pre-incubating anti-CNB antibody with purified CNB eliminates centrosomal localization. Immunofluorescence of cytosol depleted hTERT-RPE1 cells. Centrioles are marked by anti-POC5 staining (green) and nuclei are marked by DAPI (blue). CNB localization (red) is analyzed by staining cells with anti-CNB antibody that has been pre-incubated with purified proteins transferred onto a nitrocellulose membrane as shown in A. In the top panels, anti-CNB antibody was incubated with bovine serum albumin (BSA) in two independent experiments. In the bottom panels, anti-CNB antibody was incubated with CNB in two independent experiments. Lines were drawn across the two centrioles of each cell (shown in yellow) to generate line intensity plots on the right of each immunofluorescence panel. Line intensity plots track the intensity of CNB signal (red) and POC5 signal (green) across the cell and the two centrioles, indicated by the double peaks of POC5 intensity. Scale bar, 5 μm.

Journal of Cell Science • Supplementary information

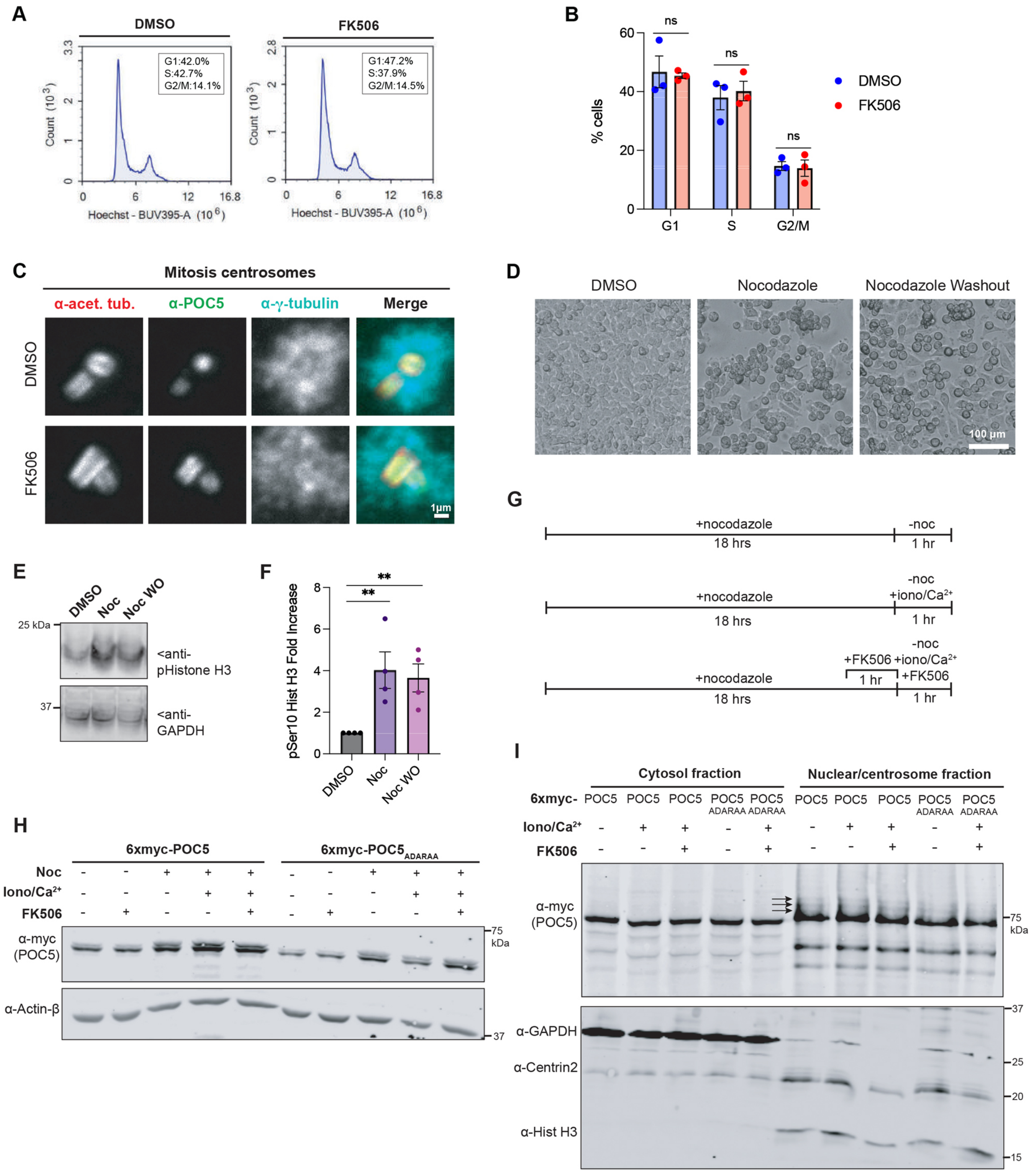

Fig. S3 (Related to Figure 3). Calcineurin activity disrupts POC5 distribution, but does not alter γ-tubulin distribution or POC5 phospho-status *in vivo*.

A. Cell cycle distribution of hTERT-RPE1 cells treated with DMSO or FK506 for 48 hours and analyzed by flow cytometry.

B. Statistical results of three replicates of cell cycle distribution experiments, one of which is shown in A. Bars represent the mean ± SEM. p-values > 0.05 determined by paired, two-tailed t-test.

Journal of Cell Science • Supplementary information

C. Expansion microscopy of mitotic hTERT-RPE1 cells treated with DMSO or FK506 for 48 hours, showing POC5 in the centriole lumen and γ-tubulin in the lumen and PCM. Images obtained from maximum projections of confocal z-stacks. Scale bar, 1 μm.

D. Samples are enriched for mitotic cells 1 hour post nocodazole washout. Brightfield microscopy images showing HeLa cells that are asynchronous (DMSO), immediately after synchronization with nocodazole, and 1 hour after nocodazole washout. Mitotic cells are rounded up. Scale bar, 100 μm.

E. Immunoblot of HeLa cell lysates treated with DMSO (control), immediately after 18 hours of treatment with nocodazole, or 1 hour after nocodazole washout.

F. Quantification of phosphorylated Histone H3 expression from immunoblot shown in F. Data represent anti-pSer Histone H3 signal normalized to GAPDH loading control, relative to expression under DMSO treatment. Bars represent the mean ± SEM (n=3 independent experiments). n.s., not significant, *p < 0.05, **p < 0.01, ***p < 0.001. p-values determined by ratio-paired, two-tailed t-test. For F,G: Noc, nocodazole. Noc WO, nocodazole washout.

G. Timeline of nocodazole synchronization combined with CN activation or inhibition for experiments shown in H and I. Noc, nocodazole.

H. POC5 is phosphorylated in mitosis independently of CN stimulation or inhibition. Immunoblot showing lysates of HeLa cells transfected with 6xmyc-POC5 or -$POC5_{ADARAA}$. Cells were treated with DMSO (-) or 100 ng nocodazole (+) for 18 hours and then incubated for one additional hour in 37°C after drug washout. The second POC5 band that appears in nocodazole (+) samples corresponds to p-POC5. For CN activation, samples were additionally treated with 1 μM ionomycin + 1 mM $CaCl_2$ for one hour prior to cell lysis. For CN inhibition, samples were treated with 2.5 μM FK506 for one hour followed by 2.5 μM FK506 + 1 μM ionomycin + 1 mM $CaCl_2$ for one more hour prior to cell lysis. Iono, ionomycin. Ca^{2+}, calcium ions from CaCl2 addition. Anti-actin-beta was used as a loading control. Noc, nocodazole.

I. POC5 is hyperphosphorylated at centrosomes independently of CN stimulation or inhibition. Cytosolic and nuclear-centrosomal fractions prepared via sucrose fractionation from HeLa cells transfected with 6xmyc-POC5 or -$POC5_{ADARAA}$. For CN activation, samples were treated with 1 μM ionomycin + 1 mM $CaCl_2$ for one hour prior to fractionation. For CN inhibition, samples were treated with 2.5 μM FK506 for one hour followed by 2.5 μM FK506 + 1 μM ionomycin + 1 mM $CaCl_2$ for one more hour prior to fractionation. Iono, ionomycin. Ca^{2+}, calcium ions from $CaCl_2$ addition. Anti-GAPDH was used a cytosolic marker, anti-centrin2 as a centrosomal marker and anti-histone H3 as a nuclear marker.

Journal of Cell Science • Supplementary information

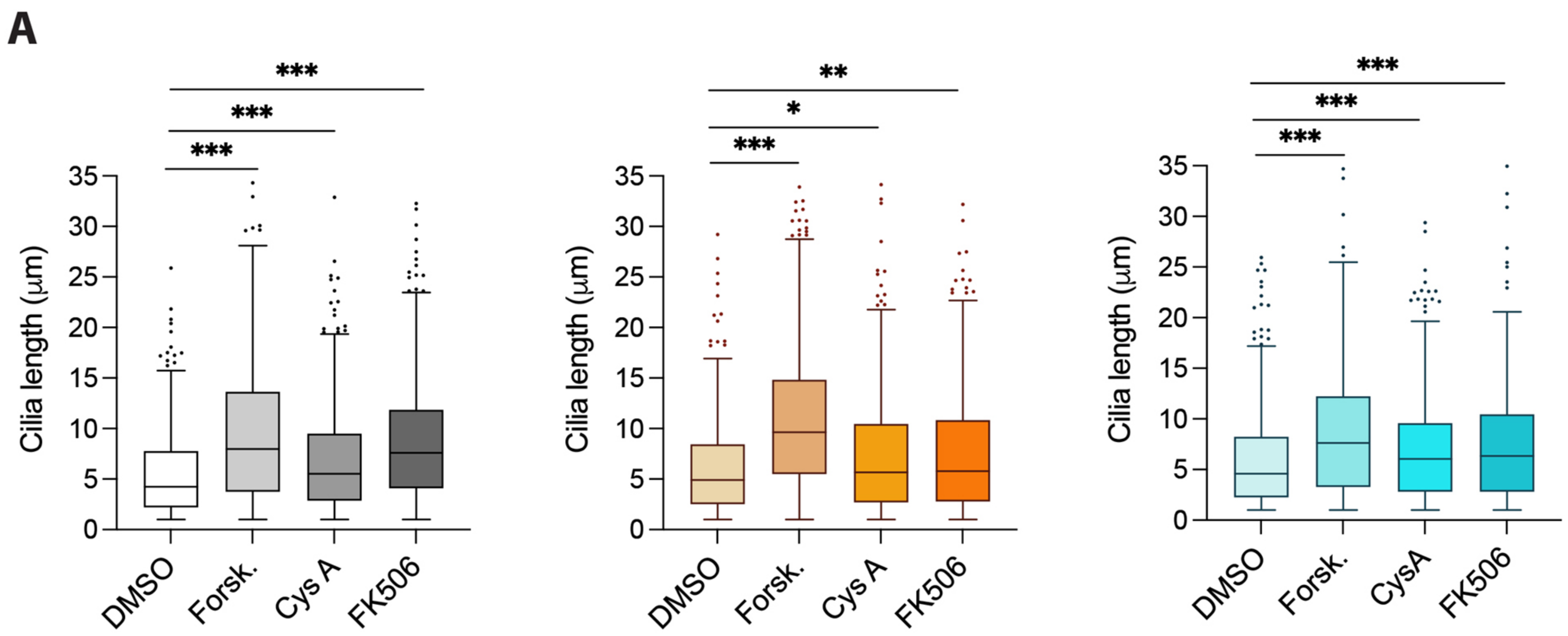

Fig. S4. (Related to Figure 4) Calcineurin inhibition increases cilia length.

A. Forskolin treatment and CN inhibition consistently promote cilia elongation. Cilia length in ciliated IMCD3 cells treated for 3 hours with DMSO, forskolin, cyclosporin A or FK506 as in Fig. 4A. Graphs show data from three independent experimental replicates in addition to the replicate shown in 4B. Cilia length was determined by 3D vector analysis of confocal z-stacks using CiliaQ with CANNY 3D segmentation (Hansen et al., 2021). Only continuous Arl13b branches with length > 1 µm are shown on the graph. Number of cilia measured: leftmost graph, DMSO, n=475, forskolin, n=564, cyclosporin A, n=481, FK506, n=718. Center graph, DMSO, n=497, forskolin, n=602, cyclosporin A, n=603, FK506, n=494. Rightmost graph, DMSO, n=371, forskolin, n=417, cyclosporin A, n=421, FK506, n=470. Boxplots show median length ± interquartile range (IQR) and whiskers represent the median ± 1.5 x IQR. n.s., not significant, *$p < 0.05$, **$p < 0.01$, ***$p < 0.001$. p-values determined using two-tailed Mann-Whitney test.

Journal of Cell Science • Supplementary information

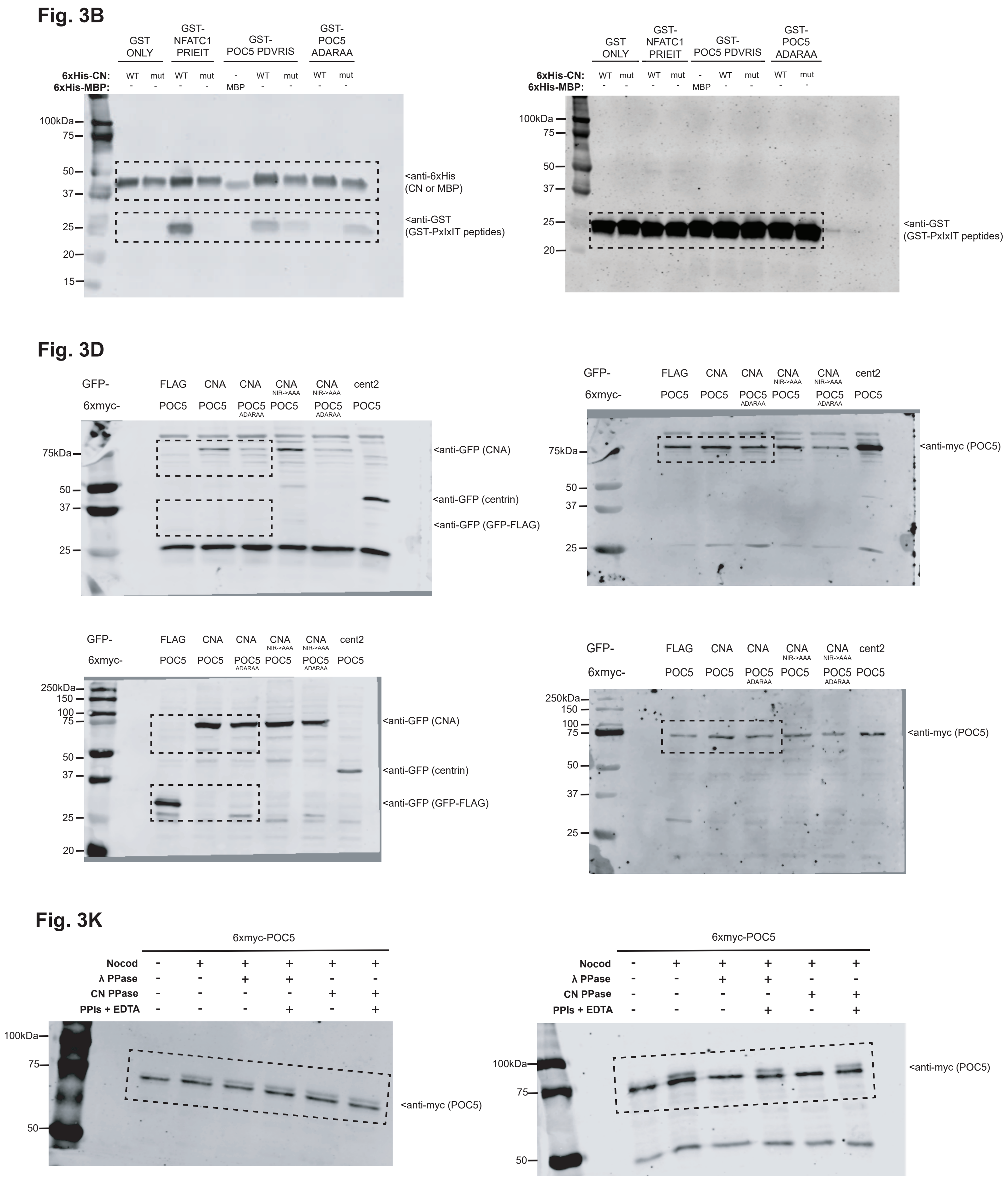

Fig. S5. Uncropped western blots from Figure 3.

Journal of Cell Science • Supplementary information

Fig. S1A

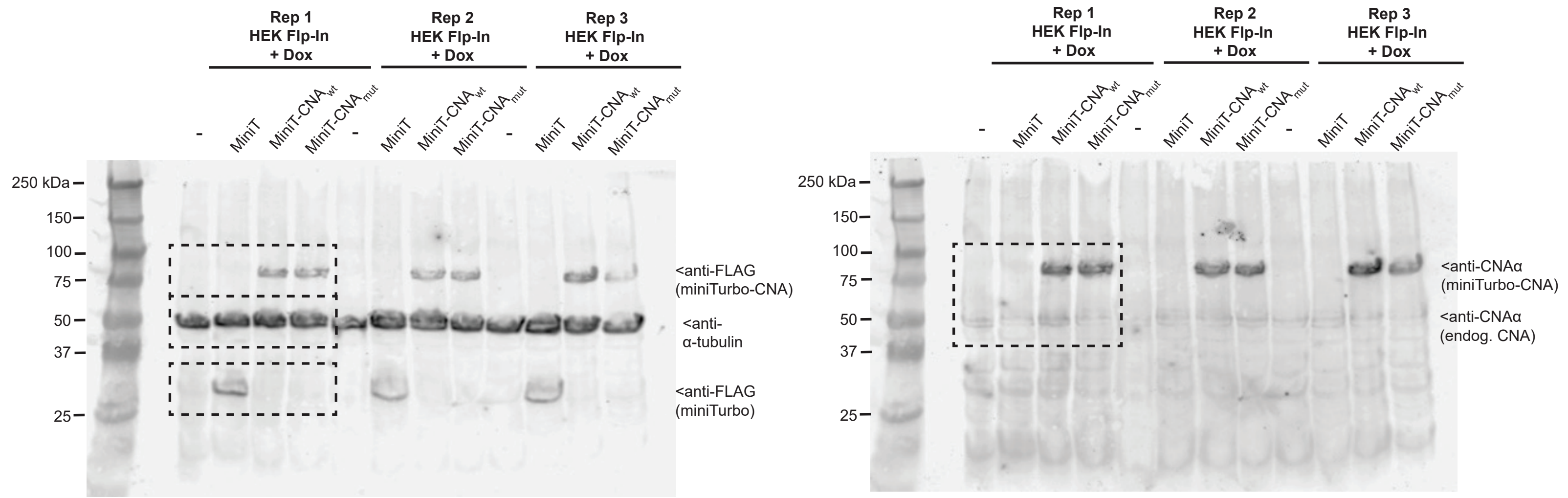

Fig. S1E

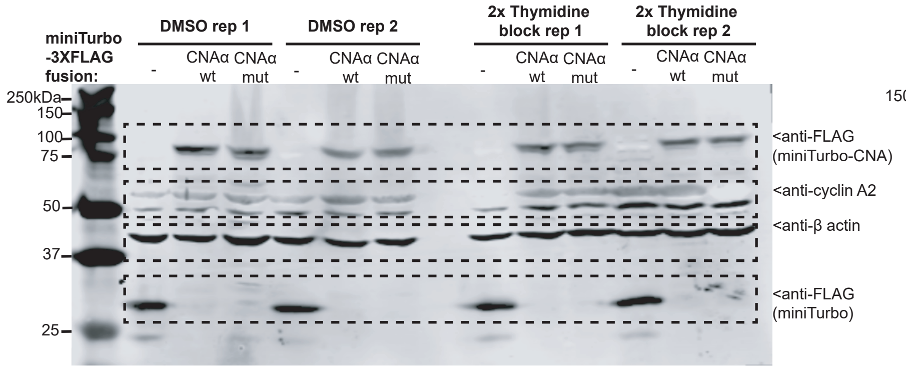

Fig. S1F

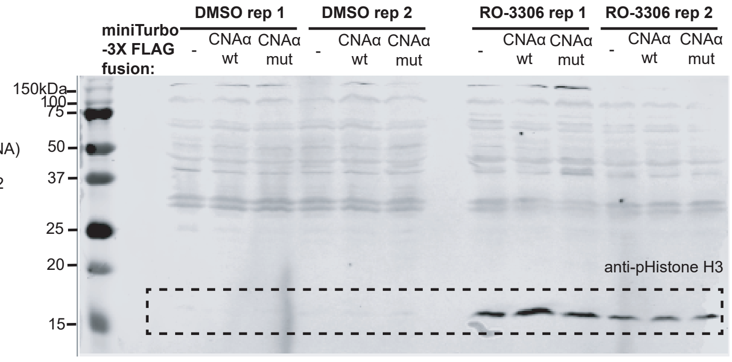

Fig. S1F

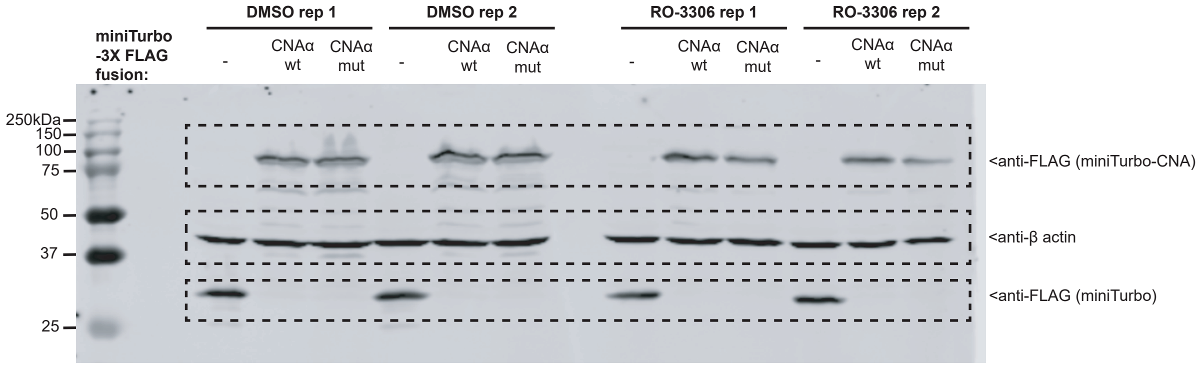

Fig. S2A

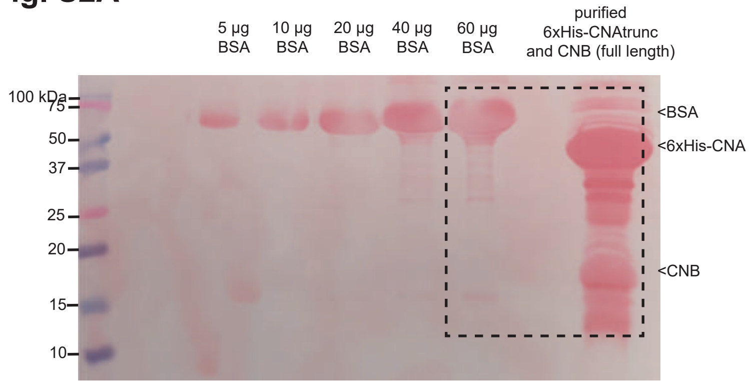

Journal of Cell Science • Supplementary information

Fig. S3E

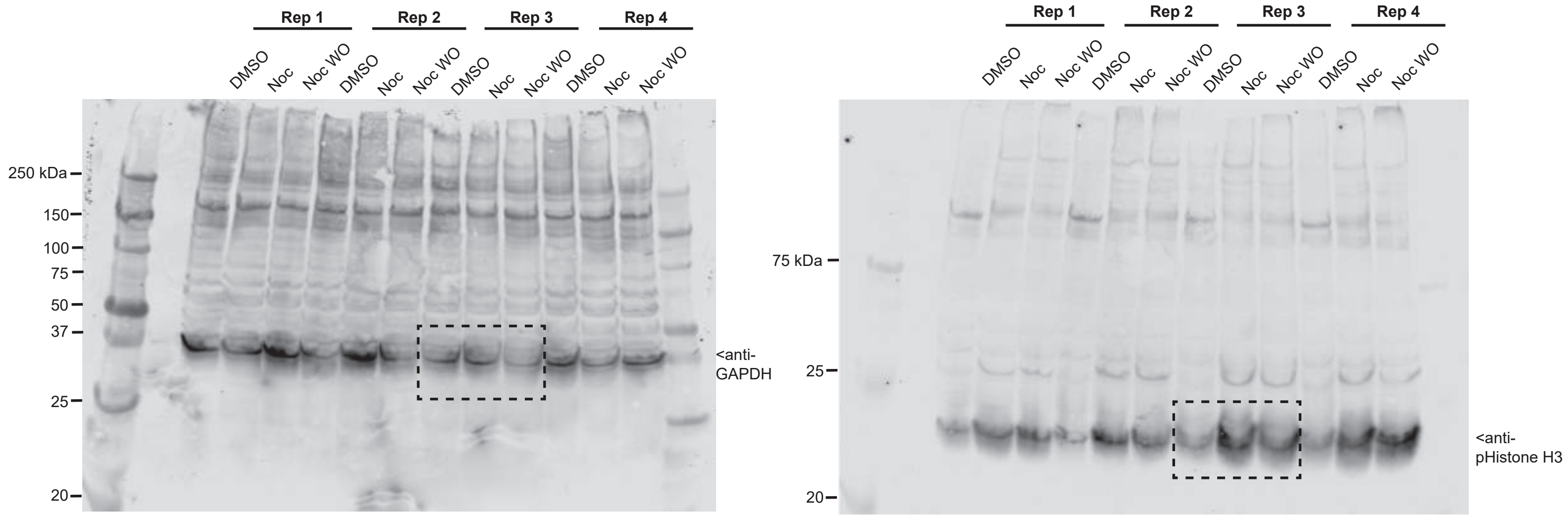

Fig. S3H

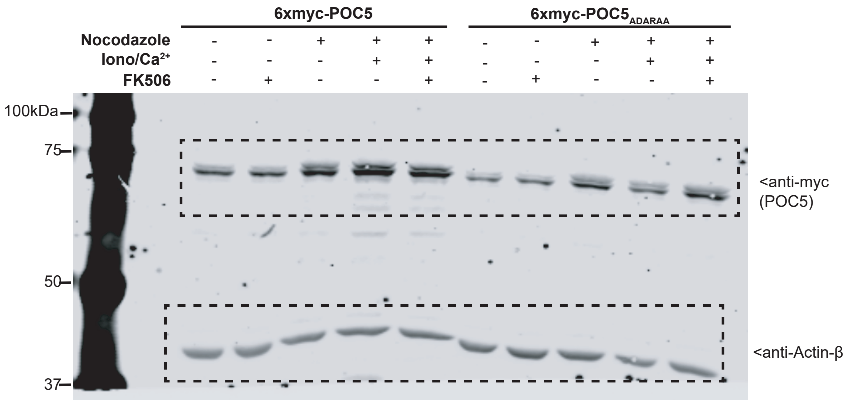

Fig. S3I

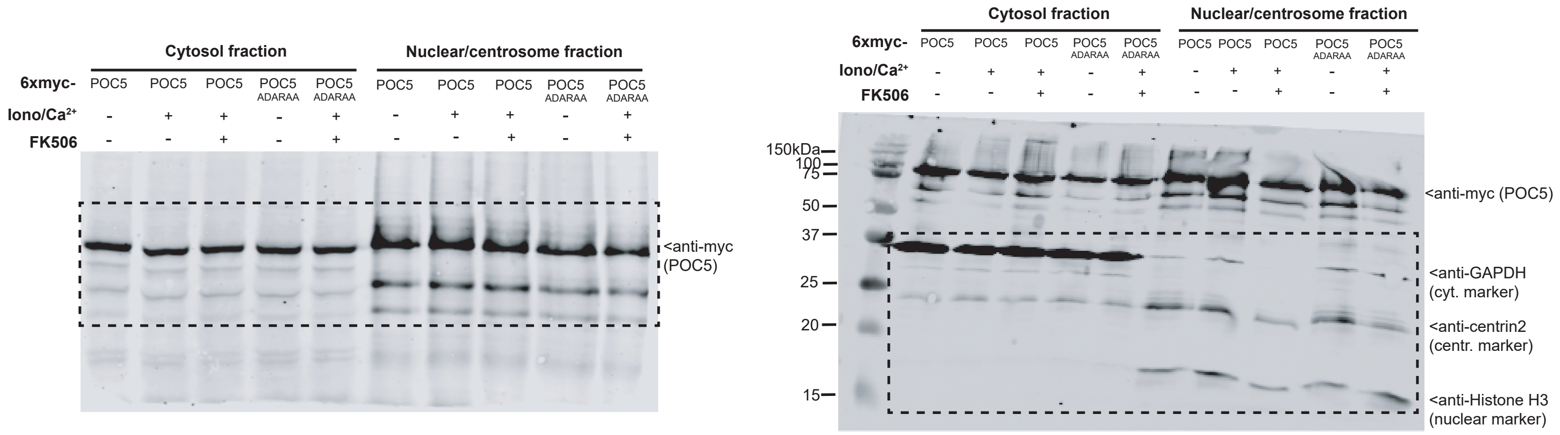

Fig. S6. Uncropped western blots from Supplemental Figures.

Journal of Cell Science • Supplementary information

Table S1. Dataset of proteins identified via PDB-MS, final filtered dataset and associated GO term analyses.

MiniTurbo-MS data-dependent acquisition (DDA) dataset analyzed by SAINTexp3.6.1 and data-independent mixture-spectrum partitioning using libraries of identified tandem mass spectra (mSPLIT) dataset analyzed by SAINTexp3.6.3, filtered DDA dataset and associated GO Term analyses.

Click here to download Table S1

Table S2. Subcellular localization of centrosome-associated, calcineurin-proximal proteins.

Table of relevant literature regarding cilia and centrosome components that were proximal to miniTurbo-CN in our study. Proteins are identified by their corresponding gene name, with a brief summary of their precise subcellular location as described by the referenced studies.

Gene name	Protein localization	References
AKAP5 (AKAP79, AKAP150)	Cilia, centrosomes, spindle poles	(Choi et al., 2011)
ALMS1	Proximal centrioles	(Knorz et al., 2010)
CDC16	Centrosomes and spindle poles	(Tugendreich et al., 1995)
CEP78	Distal centrioles	(Hossain et al., 2017)
CEP97	Distal centrioles	(Spektor et al., 2007)
EXOC4 (SEC8)	Cilia, centrosomes	(Gupta et al., 2015; Zuo et al., 2011)
GAK	Spindle poles during mitosis	(Fukushima et al., 2017; Naito et al., 2012)
HAUS5	Centrosomes in interphase, mitotic spindle microtubules, middle to distal centrioles	(Lawo et al., 2009; Schweizer et al., 2021)
HOOK1	Broad distribution around centrosomes	(Szebenyi et al., 2007)
POC5	Middle to distal centrioles	(Le Guennec et al., 2020)
PPP1R35	Proximal centrioles	(Sydor et al., 2018)
SMC1A	Centrioles, spindle poles	(Guan et al., 2008; Wong, 2010)
SPICE1	Proximal centrioles (distal to SAS-6, proximal to centrin)	(Comartin et al., 2013)
TALPID3 (KIAA0586)	Distal centrioles	(Kobayashi et al., 2014)

Journal of Cell Science • Supplementary information

Supplemental References

Comartin, D., Gupta, G. D., Fussner, E., Coyaud, É., Hasegan, M., Archinti, M., Cheung, S. W. T., Pinchev, D., Lawo, S., Raught, B., et al. (2013). CEP120 and SPICE1 cooperate with CPAP in centriole elongation. *Curr. Biol.* **23**, 1360–1366.

Fukushima, K., Wang, M., Naito, Y., Uchihashi, T., Kato, Y., Mukai, S., Yabuta, N. and Nojima, H. (2017). GAK is phosphorylated by c-Src and translocated from the centrosome to chromatin at the end of telophase. *Cell Cycle* **16**, 415–427.

Guan, J., Ekwurtzel, E., Kvist, U. and Yuan, L. (2008). Cohesin protein SMC1 is a centrosomal protein. *Biochem. Biophys. Res. Commun.* **372**, 761–764.

Gupta, G. D., Coyaud, É., Gonçalves, J., Mojarad, B. A., Liu, Y., Wu, Q., Gheiratmand, L., Comartin, D., Tkach, J. M., Cheung, S. W. T., et al. (2015). A dynamic protein interaction landscape of the human centrosome-cilium interface. *Cell* **163**, 1484–1499.

Hossain, D., Javadi Esfehani, Y., Das, A. and Tsang, W. Y. (2017). Cep78 controls centrosome homeostasis by inhibiting EDD-DYRK2-DDB1VprBP. *EMBO Rep.* **18**, 632–644.

Knorz, V. J., Spalluto, C., Lessard, M., Purvis, T. L., Adigun, F. F., Collin, G. B., Hanley, N. A., Wilson, D. I. and Hearn, T. (2010). Centriolar association of ALMS1 and likely centrosomal functions of the ALMS motif–containing proteins C10orf90 and KIAA1731. *Mol. Biol. Cell* **21**, 3617–3629.

Kobayashi, T., Kim, S., Lin, Y.-C., Inoue, T. and Dynlacht, B. D. (2014). The CP110-interacting proteins Talpid3 and Cep290 play overlapping and distinct roles in cilia assembly. *J. Cell Biol.* **204**, 215–229.

Lawo, S., Bashkurov, M., Mullin, M., Ferreria, M. G., Kittler, R., Habermann, B., Tagliaferro, A., Poser, I., Hutchins, J. R. A., Hegemann, B., et al. (2009). HAUS, the 8-subunit human Augmin complex, regulates centrosome and spindle integrity. *Curr. Biol.* **19**, 816–826.

Naito, Y., Shimizu, H., Kasama, T., Sato, J., Tabara, H., Okamoto, A., Yabuta, N. and Nojima, H. (2012). Cyclin G-associated kinase regulates protein phosphatase 2A by phosphorylation of its B'γ subunit. *Cell Cycle* **11**, 604–616.

Szebenyi, G., Hall, B., Yu, R., Hashim, A. I. and Krämer, H. (2007). Hook2 localizes to the centrosome, binds directly to centriolin/CEP110 and contributes to centrosomal function. *Traffic* **8**, 32–46.

Tugendreich, S., Tomkiel, J., Earnshaw, W. and Hieter, P. (1995). CDC27Hs colocalizes with CDC16Hs to the centrosome and mitotic spindle and is essential for the metaphase to anaphase transition. *Cell* **81**, 261–268.

Wong, R. W. (2010). Interaction between Rae1 and cohesin subunit SMC1 is required for proper spindle formation. *Cell Cycle* **9**, 198–200.

Zuo, X., Fogelgren, B. and Lipschutz, J. H. (2011). The small GTPase Cdc42 is necessary for primary ciliogenesis in renal tubular epithelial cells. *J. Biol. Chem.* **286**, 22469–22477.

Journal of Cell Science • Supplementary information
