# Supplementary material for: Calcineurin associates with centrosomes and regulates cilia length maintenance
Source: J Cell Sci. 2023 Apr 24;136(8):jcs260353. doi: 10.1242/jcs.260353 (PMC10163345; doi:10.1242/jcs.260353)
Supplement: Supplementary file 1 [file joces-136-260353_review_history.pdf]

Calcineurin associates with centrosomes and regulates cilia length maintenance

Eirini Tsekitsidou, Cassandra J. Wong, Idil Ulengin-Talkish, Angela I. M. Barth, Tim Stearns, Anne-Claude Gingras, Jennifer T. Wang and Martha S. Cyert
DOI: 10.1242/jcs.260353

Editor: David Glover

Review timeline

Original submission:	18 June 2022
Editorial decision:	1 September 2022
First revision received:	4 January 2023
Editorial decision:	20 February 2023
Second revision received:	21 March 2023
Accepted:	27 March 2023

Original submission

First decision letter

MS ID#: JOCES/2022/260353

MS TITLE: Calcineurin associates with centrosomes and regulates cilia length maintenance

AUTHORS: Eirini Tsekitsidou, Jennifer T Wang, Cassandra J Wong, Idil Ulengin-Talkish, Tim Stearns, Anne-Claude Gingras, and Martha S. Cyert
ARTICLE TYPE: Short Report

We have now reached a decision on the above manuscript.

To see the reviewers' reports and a copy of this decision letter, please go to: https://submit-jcs.biologists.org and click on the 'Manuscripts with Decisions' queue in the Author Area. (Corresponding author only has access to reviews.)

As you will see, the reviewers raise a number of substantial criticisms that prevent me from accepting the paper at this stage. They suggest, however, that a revised version might prove acceptable, if you can address their concerns. If you think that you can deal satisfactorily with the criticisms on revision, I would be pleased to see a revised manuscript. We would then return it to the reviewers.

Please ensure that you clearly highlight all changes made in the revised manuscript. Please avoid using 'Tracked changes' in Word files as these are lost in PDF conversion.

I should be grateful if you would also provide a point-by-point response detailing how you have dealt with the points raised by the reviewers in the 'Response to Reviewers' box. Please attend to all of the reviewers' comments. If you do not agree with any of their criticisms or suggestions please explain clearly why this is so.

Reviewer 1

Advance summary and potential significance to field

© 2023. Published by The Company of Biologists under the terms of the Creative Commons Attribution License (https://creativecommons.org/licenses/by/4.0/).

The study by Tsekitsidou et al. investigates the potential role of calcineurin, a calcium-regulated phosphatase, at centrioles. This was prompted by the discovery of various centrosome and ciliary proteins in proximity labeling experiments in human cells at different cell cycle stages using calcineurin as bait. While the authors did not find major cell cycle-related differences, they confirmed earlier reports of proximity interactions of calcineurin with centrosome proteins. They also found that many of these interactions were dependent on the presence of a calcineurin substrate motif (PxIxIT-type). Consistently, the authors show that calcineurin localizes to centrioles, suggesting that the interactions may take place at these organelles. The authors further demonstrate that one of these centriole proteins, POC5, interacts with calcineurin in vitro and in cells in a PxIxIT-dependent manner. Finally, the authors show that chemical inhibition of calcineurin alters POC5 distribution in the centriole lumen and induces elongation of primary cilia in cultured cells.
Overall this is a solid study that establishes a role of calcineurin at centrioles and in particular in ciliary length control, linking this process with calcium signaling. Most data is of high quality and well-presented. A weak point is the missing link between calcineurin-mediated POC5 centriolar distribution and the ciliary length phenotype. However, if the authors address a few issues, the manuscript may still provide interesting and important advance.

Comments for the author

Specific points:

1) Fig. 3F and S3B: Recombinant POC5 WT and mutant expression: It is possible that the presence of endogenous POC5 may mask any localization defects (3F) or altered phosphorylation (S3B) of mutant POC5 (e.g. if POC5 oligomerizes, etc).
These experiments should be performed in POC5 RNAi cells and localization should be analyzed by U-ExM. Reg. localization, if this is difficult in RPE1 due to cell cycle arrest, it could also be performed in another cell line such as U2OS.

2) S3B: why were the cells released from mitosis by noc washout? Also according to the legend, for the different treatments different incubations times were used following washout. This does not allow a reliable comparison between samples. Moreover, it seems that mitotic cells were not specifically analyzed by shake-off. It may be worth synchronizing cells in mitosis with noc and then without release, perform the treatments for the same duration. For analysis harvesting only mitotic cells by shake-off, may also be beneficial.

3) Fig. 3G-I: The U-ExM analysis is nice, but the quality of the POC5 stainings is not sufficient to illustrate the claimed distribution defect in inhibitor-treated cells. POC5 localizes to the inner centriole wall as previously shown by others and as seen in the control cells in 3G (2 parallel lines in centriole side-view). In the example image of the FK506 condition POC5 does not seem to line the inner wall anymore, but rather seems to localize more luminal (2 parallel lines cannot be distinguished). Is this indeed the case? If so, the authors should come up with a better way of quantifying this.

4) Related to point 2 above, does FK506 treatment affect cell cycle progression in RPE1? G1 arrest, for example, may mask defects in POC5 luminal recruitment, which occurs in S/G2. Again, testing this in another cell line, may be an option.

5) S3A, B; related to point 4: once incorporated into the centriole lumen POC5 may be quite stable. Thus, it may indeed be informative to look at the effects of calcineurin inhibition specifically in newly formed daughter centrioles in late S/G2 that have only recently acquired luminal POC5. The G2 phase images in S3A are likely late S phase, since daughters are still very short (based on acet. tubulin) and contain hardly any POC5 even in controls.

Reviewer 2

Advance summary and potential significance to field

In this paper, Tsekitsidou et al. identified the first cell cycle-linked proximity interactome of calcineurin (CN) and found that it does not change drastically. By focusing on the centrosome

© 2023. Published by The Company of Biologists under the terms of the Creative Commons Attribution License (https://creativecommons.org/licenses/by/4.0/).

proteins enriched in CN proximity interactomes, they then defined CN as a centrosome protein and identified POC5 as a direct interactor of CN. Finally, inhibitor treatment experiments in IMCD3 cells revealed CN functions in cilium length regulation.

Given the critical functions associated with CN in signaling and its use as a target for immunosuppression these findings provide an important advance in our understanding of the multifaceted functions of CN at different subcellular locations and are also of clinical relevance. Wigington et al. 2020 paper from the Cyert lab previously identified the proximity interactome of CN using the BioID enzyme, which is limited in temporal resolution. The datasets from the 2020 paper also reported the extensive proximity interactions of CN with centrosome proteins. Therefore, the identification of CN proximity partners and centrosomal association is an incremental advance. However, its function in cilium length regulation is novel and will lead to further studies in uncovering the mechanisms and disease links of this function. Moreover, the manuscript is very well-written. I thank the authors for the extensive detail they provide in the methods section.

Comments for the author

Below are my comments on the manuscript, which will strengthen their conclusions and provide more insight into CN functions linked to its centrosome pool:

1- What is the localization and biotinylation profile of the miniTurbo-CN wild-type and mutant during the cell cycle stages used for identification of proximity maps? Do they localize to the centrosome and induce biotinylation at the centrosomes?

2- For the lines used in proteomics experiments, how much are the fusion proteins overexpressed relative to the endogenous proteins? Lower expression relative to endogenous protein might explain the low spectral counts of baits (in addition to short biotinylation time)

3- Since the authors indicate that 23/41 preys were common to asynchronous, G1/S and mitotic populations, this means that about half of the preys are not common. Doesn't this suggest that CN spatial distribution is not constant throughout the cell cycle?

4- The authors provide more detail on the manual curation of the literature (which datasets, resources?) to identify 14 CN-proximal proteins that distribute throughout the centrosome/cilium and/or localize to the mitotic spindle (Fig. 1D).

5- It is interesting that the authors reported two different localization profiles for CNB at the centrosome (overlapping with centrioles and between centrioles). Is this variation correlate with different cell cycle stages? Does tagged CNB (fluorescent protein or a small tag) exhibit similar localization profile? This can be checked in the HEK293T stable lines they generated for streptavidin pulldowns?

6- The representative U-ExM images for CNB in Fig. 2B confirms centriole localization, but it is hard to distinguish between centriole lumen and wall localization based on these images. Deconvolution of the U-ExM data might help resolve this issue. Given that this data is the first report of CN localization to the centriole localization analysis relative to previously characterized centriole inner core proteins other than POC5 will inform on the function of CN at the centrioles and strengthen the conclusions on CN's localization at the centriole (similar to how WDR90 was analyzed in Steib et al., 2021 paper).

7- Since POC5 abundance at the centriole is affected upon CN inhibition, the authors should elucidate whether CN inhibition also affects cellular abundance of POC5 or not. Moreover, does CN inhibition affect centriolar abundance of other centriole core/lumen proteins (i.e. centrin, POC1B, FAM161A)?

8- Do the cilia that form in CN-inhibited cells functional (does it respond to Hedgehog stimuli)? Does overexpression of CN affect cilium length antagonistically to its inhibition?

9- The authors focused on characterization of cilia phenotypes associated with CN inhibition. Given the previously described functions of POC5 and centriole lumen/core proteins in centriole

© 2023. Published by The Company of Biologists under the terms of the Creative Commons Attribution License (https://creativecommons.org/licenses/by/4.0/).

integrity, the authors should also address CN's role in centriole integrity by quantifying centriole length and structural defects using expansion microscopy.

First revision

Author response to reviewers' comments

Tsekitsidou et al., Point by Point response to reviewers' comments:

Reviewer 1
Advance Summary and Potential Significance to Field:
The study by Tsekitsidou et al. investigates the potential role of calcineurin, a calcium-regulated phosphatase, at centrioles. This was prompted by the discovery of various centrosome and ciliary proteins in proximity labeling experiments in human cells at different cell cycle stages using calcineurin as bait. While the authors did not find major cell cycle-related differences, they confirmed earlier reports of proximity interactions of calcineurin with centrosome proteins. They also found that many of these interactions were dependent on the presence of a calcineurin substrate motif (PxIxIT-type). Consistently, the authors show that calcineurin localizes to centrioles, suggesting that the interactions may take place at these organelles. The authors further demonstrate that one of these centriole proteins, POC5, interacts with calcineurin in vitro and in cells in a PxIxIT-dependent manner. Finally, the authors show that chemical inhibition of calcineurin alters POC5 distribution in the centriole lumen and induces elongation of primary cilia in cultured cells.
Overall this is a solid study that establishes a role of calcineurin at centrioles and in particular in ciliary length control, linking this process with calcium signaling. Most data is of high quality and well-presented. A weak point is the missing link between calcineurin-mediated POC5 centriolar distribution and the ciliary length phenotype. However, if the authors address a few issues, the manuscript may still provide interesting and important advances.

We thank the reviewer for acknowledging the high quality of our data and the advances in knowledge demonstrated by our study.

Specific points:

1) Fig. 3F and S3B: Recombinant POC5 WT and mutant expression: It is possible that the presence of endogenous POC5 may mask any localization defects (3F) or altered phosphorylation (S3B) of mutant POC5 (e.g. if POC5 oligomerizes, etc). These experiments should be performed in POC5 RNAi cells and localization should be analyzed by U-ExM. Reg. localization, if this is difficult in RPE1 due to cell cycle arrest, it could also be performed in another cell line such as U2OS.

We thank the reviewer for this comment. However, we'd like to emphasize that our goal was to determine if the mutant POC5 protein lacking the PxIxIT was capable of being recruited to centrosomes, which the current analyses demonstrate. Rather than carrying out siRNA experiments, which as Azimzadeh et al 2009 showed takes several cell divisions to effect depletion and required deletion of p53, we have clarified the text to acknowledge this caveat to our current experiment. The text now reads "we carried out indirect immunofluorescence of cells transiently expressing 6xmyc-POC5 (POC5WT or mutated PxIxIT POC5ADARAA) in the presence of endogenous POC5". We also changed figure 3 figure legend to read, "F. Transiently expressed 6xmyc-POC5WT or POC5ADARAA is recruited to centrosomes.

2) S3B: why were the cells released from mitosis by noc washout? Also, according to the legend, for the different treatments different incubations times were used following washout. This does not allow a reliable comparison between samples. Moreover, it seems that mitotic cells were not specifically analyzed by shake-off. It may be worth synchronizing cells in mitosis with noc and then, without release, perform the treatments for the same duration. For analysis, harvesting only mitotic cells by shake-off, may also be beneficial.

© 2023. Published by The Company of Biologists under the terms of the Creative Commons Attribution License (https://creativecommons.org/licenses/by/4.0/).

As noted by the reviewer, to capture POC5 mitotic phosphorylation, we arrested cells in G2/M by incubating with nocodazole, followed by a brief wash out. We have now added panels D-F to Figure S3A, showing that after this 1 hour wash out, the culture is in fact enriched in mitotic cells as shown by their rounded cell morphology, and a strong immunoblot signal with a mitosis- specific antibody (i.e. anti phosphorylated histone H3). Also, the phosphorylated status of POC5 we observed recapitulates the findings by Azimzadeh et al (2009). We opted to use this approach rather than mitotic shake-off because of the fairly large amount of material needed to carry out immunoprecipitation. Finally, we apologize that the timing of the different drug treatments was confusing. We have now added a schematic to Figure S3 (panel G) to clarify the protocol: All samples were incubated with nocodazole for the same amount of time, i.e. 18 hr, and in each case were harvested 1 hour after washing out nocodazole, but in the presence of Ca2+, ionomycin and/or FK506 as indicated.

3) Fig. 3G-I: The U-ExM analysis is nice, but the quality of the POC5 stainings is not sufficient to illustrate the claimed distribution defect in inhibitor-treated cells. POC5 localizes to the inner centriole wall as previously shown by others and as seen in the control cells in 3G (2 parallel lines in centriole side-view). In the example image of the FK506 condition POC5 does not seem to line the inner wall anymore, but rather seems to localize more luminal (2 parallel lines cannot be distinguished). Is this indeed the case? If so, the authors should come up with a better way of quantifying this.

We understand the reviewer’s concern. In fact, the vast majority of our images of control or FK506-treated centrioles do show two parallel lines of POC5 staining in the centriole side-view, although occasionally the degree of expansion achieved was not quite sufficient to observe decreased staining of the centriole lumen. We have updated Fig. 3G, which now shows a single centriole from control or FK506-treated cells, with the maximum expansion factor achieved, that better represents our results.

For measurements of % centriole coverage by POC5 and γ-tubulin (Figs 3H and I) we divided the length of the centriole marked by POC5/ γ-tubulin over the length covered by acetylated tubulin. Since these two measurements occurred within the same centriole, the same expansion factor applies to both. For Fig. 3J, where we only measured centriole length based on acetylated tubulin signal, we excluded centrioles that were not sufficiently expanded (meaning a darker lumen was not visible), to avoid less expanded centrioles artificially skewing measurements. All measurements were performed on single z-plane images exactly as described in Schweizer et al. 2021, *Nature Communications*: *“Only centrioles with the longitudinal axis parallel to the xy plane were analysed. The lengths were measured using a line scan drawn along the longitudinal axis in the middle of the centrioles (at mid-width and on a single z section at mid-height). The centriole length was measured based on the acetylated a- tubulin staining.”* This is also stated in the Methods section.

4) Related to point 2 above, does FK506 treatment affect cell cycle progression in RPE1? G1 arrest, for example, may mask defects in POC5 luminal recruitment, which occurs in S/G2. Again, testing this in another cell line, may be an option.

In answer to the reviewer’s question, FK506 treatment does not affect cell cycle progression of hTert-RPE1 cells. We have included FACS analyses in Figs S3A and B that demonstrate this point and refer to this point in the text.

5) S3A, B; related to point 4: once incorporated into the centriole lumen, POC5 may be quite stable. Thus, it may indeed be informative to look at the effects of calcineurin inhibition specifically in newly formed daughter centrioles in late S/G2 that have only recently acquired luminal POC5. The G2 phase images in S3A are likely late S phase, since daughters are still very short (based on acet. tubulin) and contain hardly any POC5 even in controls.

We thank the reviewer for the suggestion that newly formed centrioles may be particularly sensitive to calcineurin inhibition. An image of mitotic centrioles has been added to Figure S3C, and we examined all centrioles in our data set that were either in G2 or mitosis, when the procentriole is a bit longer. However, we determined that POC5 distribution could only be reliably measured in

© 2023. Published by The Company of Biologists under the terms of the Creative Commons Attribution License (https://creativecommons.org/licenses/by/4.0/).

parental centrioles, which were used to determine the effect of FK506 on POC5 distribution shown in Figure 3H.

Reviewer 2 Advance Summary and Potential Significance to Field:
In this paper, Tsekitsidou et al. identified the first cell cycle-linked proximity interactome of calcineurin (CN)and found that it does not change drastically. By focusing on the centrosome proteins enriched in CN proximity interactomes, they then defined CN as a centrosome protein and identified POC5 as a direct interactor of CN. Finally, inhibitor treatment experiments in IMCD3 cells revealed CN functions in cilium length regulation.

Given the critical functions associated with CN in signaling and its use as a target for immunosuppression, these findings provide an important advance in our understanding of the multifaceted functions of CN at different subcellular locations and are also of clinical relevance. Wigington et al. 2020 paper from the Cyert lab previously identified the proximity interactome of CN using the BioID enzyme, which is limited in temporal resolution. The datasets from the 2020 paper also reported the extensive proximity interactions of CN with centrosome proteins. Therefore, the identification of CN proximity partners and centrosomal association is an incremental advance. However, its function in cilium length regulation is novel and will lead to further studies in uncovering the mechanisms and disease links of this function. Moreover, the manuscript is very well-written. I thank the authors for the extensive detail they provide in the methods section.

We thank the reviewer for noting the novelty of our results on calcineurin-dependent regulation of cilia length and also would like to point out that this paper is the first to show direct localization of calcineurin to centrosomes.

Reviewer 2 Comments for the Author:
Below are my comments on the manuscript, which will strengthen their conclusions and provide more insight into CN functions linked to its centrosome pool:
1- What is the localization and biotinylation profile of the miniTurbo-CN wild-type and mutant during the cell cycle stages used for identification of proximity maps? Do they localize to the centrosome and induce biotinylation at the centrosomes?

We observed cytosolic localization for calcineurin-miniTurbo fusions in the cell lines we constructed, which are highly expressed (see below). Similarly, patterns of biotinylation in these cells (examined using fluorescently labeled streptavidin) were also largely cytosolic, and did not differ significantly from biotin localization in cells expressing miniTurbo alone. In our previous work with 18 hour labeling with BirA*, centrosomal biotin localization was observed (see Wigington et al, 2020). The shorter labeling times used in this study with miniTurbo may not achieve sufficient signal over noise to all visual detection of a centrosome-enriched biotin signal.

2- For the lines used in proteomics experiments, how much are the fusion proteins overexpressed relative to the endogenous proteins? Lower expression relative to endogenous protein might explain the low spectral counts of baits (in addition to short biotinylation time).

We have included panels A and B in Fig. S1, which shows that miniTurbo-CNA fusions are expressed well above the levels of endogenous calcineurin (40-60X). Thus, we attribute the low spectral counts in our baits to the properties of the miniTurbo enzyme and reduced labeling time used.

3- Since the authors indicate that 23/41 preys were common to asynchronous, G1/S and mitotic populations, this means that about half of the preys are not common. Doesn't this suggest that CN spatial distribution is not constant throughout the cell cycle?

We call the reviewer's attention to the fact that the baits appearing in only 1 or 2 samples are also those detected with the fewest spectral counts. Thus, in our manuscript we state "Notably, the most robustly detected CN-proximal proteins were common to all three conditions (23/41). Proteins detected only in M (4/41) or G1/S (3/41) were represented by low spectral counts (≤7), possibly at the limit of detection, and had no reported cell-cycle-specific functions." Therefore, we conclude that our analyses did not reveal any reliable changes in CN-proximal proteins in these different samples.

© 2023. Published by The Company of Biologists under the terms of the Creative Commons Attribution License (https://creativecommons.org/licenses/by/4.0/).

4- The authors provide more detail on the manual curation of the literature (which datasets, resources?) to identify 14 CN-proximal proteins that distribute throughout the centrosome/cilium and/or localize to the mitotic spindle (Fig. 1D).

We refer the reviewer to Supplemental Table 2, which contains citations documenting centriole localization for the proteins so identified. We carried out manual literature searches in PubMed for each of the 41 calcineurin-proximal proteins, and noted those with published evidence of localization to the centrosome.

5- It is interesting that the authors reported two different localization profiles for CNB at the centrosome (overlapping with centrioles and between centrioles). Is this variation correlate with different cell cycle stages? Does tagged CNB (fluorescent protein or a small tag) exhibit similar localization profile? This can be checked in the HEK293T stable lines they generated for streptavidin pulldowns?

We thank the reviewer for this question about the variations in CNB staining shown in Fig. 2A. In fact, most of the cells we analyzed were in G1, and we observed all of the observed patterns in these cells. We clarified this in the manuscript text by specifying that the different localization patterns were observed independent of cell cycle phase. The variation may be due to slight differences in permeabilization/extraction of cells with digitonin prior to fixation. We did not investigate CNB localization using fusions to a fluorescent protein because these fusions are problematic. N- terminal fusions interfere with the N-terminal myristoylation of CNB and C-terminal fusions do not associate properly with the CNA subunit. The HEK293 Flp-In TRex cell lines, which expressed fusions of CNA with miniTurbo, would not be useful in localization experiments due to their high level of overexpression (see Figs S1A and B). In fact, the approach we used, i.e. to visualize endogenous CNB, using a highly specific and sensitive antibody and showing that staining is blocked by preincubation of the antisera with CNB but not BSA, is extremely rigorous and is reinforced by U-ExM images of CNB localization to centrioles, which did not utilize digitonin.

6- The representative U-ExM images for CNB in Fig. 2B confirms centriole localization, but it is hard to distinguish between centriole lumen and wall localization based on these images. Deconvolution of the U-ExM data might help resolve this issue. Given that this data is the first report of CN localization to the centriole, localization analysis relative to previously characterized centriole inner core proteins other than POC5 will inform on the function of CN at the centrioles and strengthen the conclusions on CN's localization at the centriole (similar to how WDR90 was analyzed in Steib et al., 2021 paper).

We enthusiastically thank the reviewer for this comment. We carried out deconvolution as suggested, and this greatly enhanced our ability to show that CNB localizes to centrioles, and that CNB staining overlaps with that of polyglutamylated tubulin at the wall of the centriole, rather than in the lumen as seen for POC5. These improved analyses are shown in revised Figure 2B. As the reviewer mentions, in the Steib et al 2020 eLIFE paper, WDR90 distribution was compared to several other proteins (POC1B, centrin, Fam161A, POC5). However, this also shows that the positions of centrin, Fam161A, POC1B and POC5 within the centriole lumen are highly similar. We did in fact, attempt to co-stain with antisera against CNB and WDR90, but were unsuccessful at visualizing centriolar WDR90 which (as shown in Steib et al 2020) is highly punctate.

7- Since POC5 abundance at the centriole is affected upon CN inhibition, the authors should elucidate whether CN inhibition also affects cellular abundance of POC5 or not. Moreover, does CN inhibition affect centriolar abundance of other centriole core/lumen proteins (i.e. centrin, POC1B, FAM161A)?

We thank the reviewer for this interesting question. Unfortunately, POC5 is quite low in abundance, and cannot be detected via immunoblot analysis with currently available antisera. Therefore, we were unable to determine whether inhibition of CN affects POC5 abundance, and did not investigate expression of other centriole core proteins. Furthermore, our findings that CN localizes to centrioles suggests that is acts locally at these organelles, rather than by affecting cellular pools of centriolar proteins.

© 2023. Published by The Company of Biologists under the terms of the Creative Commons Attribution License (https://creativecommons.org/licenses/by/4.0/).

8- Do the cilia that form in CN-inhibited cells functional (does it respond to Hedgehog stimuli)? Does overexpression of CN affect cilium length antagonistically to its inhibition?

We thank the reviewer for these questions, which suggest some very interesting areas for our future investigations of CN function at centrioles and cilia. However, we respectfully submit that investigations of cilia function are outside the scope of our current manuscript, which we are submitting as a short report. Similarly, given that CN activity is tightly regulated, overexpression of CN is challenging, as truncated, constitutively active CN would have to be used and shown to localize to centrioles. However, as we point out in the manuscript, "Our findings are consistent with depletion of RCAN2, a negative CN regulator that localizes to centrioles and basal bodies, causing ciliary shortening (Stevenson et al., 2018)." Deletion of RCAN2, a negative regulator of CN, would be expected to increase CN activity locally, suggesting in fact that increased levels of CN activity do act on cilium length antagonistically to its inhibition.

9- The authors focused on characterization of cilia phenotypes associated with CN inhibition. Given the previously described functions of POC5 and centriole lumen/core proteins in centriole integrity, the authors should also address CN's role in centriole integrity by quantifying centriole length and structural defects using expansion microscopy.

To address the reviewer's concern, we examined the centriole length in control vs FK506- treated cells and observed no difference in length as shown in revised Figure 3 panel J. Furthermore, we failed to observe any other morphological defects, and have added a statement to this effect in the manuscript text. All measurements were performed on single z- plane images exactly as described in Schweizer et al. 2021, *Nature Communications*: *"Only centrioles with the longitudinal axis parallel to the xy plane were analysed. The lengths were measured using a line scan drawn along the longitudinal axis in the middle of the centrioles (at mid-width and on a single z section at mid-height). The centriole length was measured based on the acetylated a-tubulin staining."* This is also stated in the Methods section of our manuscript.

<u>Second decision letter</u>

MS ID#: JOCES/2022/260353

MS TITLE: Calcineurin associates with centrosomes and regulates cilia length maintenance

AUTHORS: Eirini Tsekitsidou, Cassandra J Wong, Idil Ulengin-Talkish, Angela I.M. Barth, Tim Stearns, Anne-Claude Gingras, Jennifer T Wang, and Martha S. Cyert
ARTICLE TYPE: Short Report

We have now reached a decision on the above manuscript.

To see the reviewers' reports and a copy of this decision letter, please go to: https://submit-jcs.biologists.org and click on the 'Manuscripts with Decisions' queue in the Author Area. (Corresponding author only has access to reviews.)

As you will see, the reviewers gave favourable reports but raised some critical points that will require amendments to your manuscript. I hope that you will be able to carry these out because I would like to be able to accept your paper, depending on further comments from reviewers.

Please ensure that you clearly highlight all changes made in the revised manuscript. Please avoid using 'Tracked changes' in Word files as these are lost in PDF conversion.

I should be grateful if you would also provide a point-by-point response detailing how you have dealt with the points raised by the reviewers in the 'Response to Reviewers' box. Please attend to all of the reviewers' comments. If you do not agree with any of their criticisms or suggestions please explain clearly why this is so.

© 2023. Published by The Company of Biologists under the terms of the Creative Commons Attribution License (https://creativecommons.org/licenses/by/4.0/).

Reviewer 1

Advance summary and potential significance to field

The study by Tsekitsidou et al. investigates the potential role of calcineurin, a calcium-regulated phosphatase, at centrioles. This was prompted by the discovery of various centrosome and ciliary proteins in proximity labeling experiments in human cells at different cell cycle stages using calcineurin as bait. While the authors did not find major cell cycle-related differences, they confirmed earlier reports of proximity interactions of calcineurin with centrosome proteins. They also found that many of these interactions were dependent on the presence of a calcineurin substrate motif (PxIxIT-type). Consistently, the authors show that calcineurin localizes to centrioles, suggesting that the interactions may take place at these organelles. The authors further demonstrate that one of these centriole proteins, POC5, interacts with calcineurin in vitro and in cells in a PxIxIT-dependent manner. Finally, the authors show that chemical inhibition of calcineurin alters POC5 distribution in the centriole lumen and induces elongation of primary cilia in cultured cells.
Overall this is a solid study that establishes a role of calcineurin at centrioles and in particular in ciliary length control, linking this process with calcium signaling.

Comments for the author

The authors have addressed my concerns. For the new quantifications in Fig. 3J the authors should indicate in the legend the number of experiments performed and whether the data was pooled from different experiments.

Reviewer 2

Advance summary and potential significance to field

This manuscript identified the first cell cycle-linked proximity interactome of calcineurin and defined calcineurin as a centriole protein that interacts with POC5 and regulates primary cilium length.

Comments for the author

I thank the authors for addressing majority of the major and minor points I raised. The revised version of the manuscript with new data and information strengthens the major conclusions of the paper. Here are two comments I have based on the authors response to the points I raised:
1- Since the HEK stable lines expressing CN are major tools for generating the proximity interactomes as resources for the field, it was important that the authors quantified how much fusion proteins are overexpressed relative to the endogenous proteins (Fig. S1), thank you. I still find it puzzling that there was no enrichment of localized biotinylation at the centrosome by IF in these lines even though the proximity interactomes are enriched in centrosome proteins. As part of validation of the cell lines, I recommend that authors include the immunofluorescence validation data for the stable cell lines in Fig. S1.

2- As for the low abundance of POC5 (point 7), I agree with the authors that most centriole proteins are of low abundance, yet another study detected POC5 expression by western blot (Hassan et al. PLOS One 2019), suggesting then that its expression varies among different cell lines. Although I do not expect the authors to try the antibody used in this study for this manuscript, I wanted to bring it to their attention.

© 2023. Published by The Company of Biologists under the terms of the Creative Commons Attribution License (https://creativecommons.org/licenses/by/4.0/).

Second revision

Author response to reviewers' comments

Tsekitsidou et al. revised, Point by Point response to reviewers' comments:

Reviewer 1
Comments for the Author:
The authors have addressed my concerns. For the new quantifications in Fig. 3J, the authors should indicate in the legend the number of experiments performed and whether the data was pooled from different experiments.

We thank the reviewer for pointing this out. As indicated in red font we added the following sentence to the legend for Fig. 3J, "Data pooled from two independent experiments".

Reviewer 2 Comments for the Author:
I thank the authors for addressing majority of the major and minor points I raised. The revised version of the manuscript with new data and information strengthens the major conclusions of the paper. Here are two comments I have based on the authors response to the points I raised:
1- Since the HEK stable lines expressing CN are major tools for generating the proximity interactomes as resources for the field, it was important that the authors quantified how much fusion proteins are overexpressed relative to the endogenous proteins (Fig. S1), thank you. I still find it puzzling that there was no enrichment of localized biotinylation at the centrosome by IF in these lines even though the proximity interactomes are enriched in centrosome proteins. As part of validation of the cell lines, I recommend that authors include the immunofluorescence validation data for the stable cell lines in Fig. S1.

As requested by the reviewer, we have added a new panel C to Supplemental Figure1 which shows biotin distribution (as determined by fluorescence microscopy of fixed cells incubated with Streptavidin conjugated to Alexa Fluor-594) in HEK293 cell lines over-expressing miniTurbo-3xFLAG alone or fused to -CNAαWT or -CNAαNIRmut after 15 minutes of biotin addition. The cells are also stained with an anti-centrin antibody to reveal the location of centrosomes. The images reveal varying degrees of centrosomal staining with the streptavidin probe in all of these cell lines. However, the overall biotin distribution is strikingly different in the cells expressing miniTurbo alone, which show significant levels of biotin throughout the cytoplasm and nucleus, compared to cells expressing CNA-miniturbo fusions where streptavidin staining is largely excluded from the nucleus. Although there is some observed biotin labeling of centrosomes in miniTurbo expressing cells, we remind the reviewer that overall signal in these experiments is very low due to the 15-minute incubation, and that this qualitative imaging lacks the quantitative information about biotin incorporation that is achieved by mass spectrometry analysis, where CN-proximal proteins are determined by direct comparison with labeling with by miniTurbo alone on a protein-by-protein basis. Fluorescence imaging also commonly varies, depending on the fixation conditions used, vs. direct analysis of cell lysates by mass spectrometry. Finally, we note that in Wigington et al (2020) similar experiments did show a distinct difference in centrosomal biotin labeling in BirA*- vs CNAa-BirA*- expressing cells. However, the experimental conditions were very different in use of the slower labeling BirA* coupled with much longer labeling time (18 hrs) and likely had much better signal relative to background compared to the 15 min. incubations analyzed here. Consistent with this idea, many more CN-proximal proteins were identified by MS in Wigington et al (397) compared to this study (41). Therefore, we conclude that under the experimental conditions used in this study, imaging does not accurately reflect the enrichment of CN-proximal proteins at centrosomes that is revealed by MS.

2- As for the low abundance of POC5 (point 7), I agree with the authors that most centriole proteins are of low abundance, yet another study detected POC5 expression by western blot (Hassan et al. PLOS One 2019), suggesting then that its expression varies among different cell lines. Although I do not expect the authors to try the antibody used in this study for this manuscript, I wanted to bring it to their attention.
We thank the reviewer for bringing this antibody to our attention (rabbit polyclonal POC5 antibody (abcam cat: ab188330). While we did not use this specific, antibody, we did try two different

© 2023. Published by The Company of Biologists under the terms of the Creative Commons Attribution License (https://creativecommons.org/licenses/by/4.0/).

commercial antibodies to POC5 and neither gave a sufficiently specific signal on western blots to reliably detect levels of endogenous POC5 protein.

Third decision letter

MS ID#: JOCES/2022/260353

MS TITLE: Calcineurin associates with centrosomes and regulates cilia length maintenance

AUTHORS: Eirini Tsekitsidou, Cassandra J Wong, Idil Ulengin-Talkish, Angela I.M. Barth, Tim Stearns, Anne-Claude Gingras, Jennifer T Wang, and Martha S. Cyert
ARTICLE TYPE: Short Report

I am happy to tell you that your manuscript has been accepted for publication in Journal of Cell Science, pending standard ethics checks.

© 2023. Published by The Company of Biologists under the terms of the Creative Commons Attribution License (https://creativecommons.org/licenses/by/4.0/).
